# Cardiomyocyte-enriched OTUD5 alleviates pathological myocardial hypertrophy by deubiquitinating STAT3 and modulating the STAT3–PGC-1α axis

**DOI:** 10.1038/s12276-026-01870-5

**Published:** 2026-10-16

**Authors:** Bozhi Ye, Yucheng Jiang, Zhaozheng Zheng, Yanhong Jin, Yingjie Liao, Zhihan Jia, Wante Lin, Diyun Xu, Jibo Han, Zimin Fang, Naijin Zhang, Peiren Shan, Gaojun Wu, Guang Liang

**Affiliations:** 1 Department of Geriatric Medicine, The First Affiliated Hospital of Wenzhou Medical University, Wenzhou, China; 2 Department of Cardiology, The Key Laboratory of Cardiovascular Disease of Wenzhou, The First Affiliated Hospital of Wenzhou Medical University, Wenzhou, China; 3 Chemical Biology Research Center, School of Pharmaceutical Sciences, Wenzhou Medical University, Wenzhou, China; 4 Department of Cardiology, The First Hospital of China Medical University, Shenyang, People’s Republic of China; 5 School of Pharmaceutical Sciences, Hangzhou Medical College, Hangzhou, China

**Keywords:** Ubiquitylation, Mechanisms of disease

## Abstract

Pathological cardiac hypertrophy presents a important public health challenge, particularly in aging populations, highlighting the urgent need for novel therapeutic targets. This study investigates the role and molecular mechanisms of OTUD5 in pathological cardiac hypertrophy. OTUD5 expression is greatly increased in cardiomyocytes from hypertrophic hearts. Deleting cardiomyocyte-specific OTUD5 exacerbates myocardial hypertrophy induced by angiotensin II (Ang II) and transverse aortic constriction (TAC). By contrast, targeted overexpression of OTUD5 in cardiomyocytes alleviates Ang II-induced pathological hypertrophy. Mechanistically, we demonstrate that OTUD5 regulates STAT3 activity by removing the K63-linked ubiquitin chain at lysine 283 (K283) on STAT3 through its C224 residue. This process inhibits both phosphorylation and nuclear translocation of STAT3, effectively relieving its transcriptional repression of PGC1α and enhancing mitochondrial function within cardiomyocytes. Our research uncovers a new OTUD5–STAT3–PGC1α axis that modulates mitochondrial bioenergetics, suggesting it as a potential therapeutic target for pathological cardiac hypertrophy.

**Figure:**
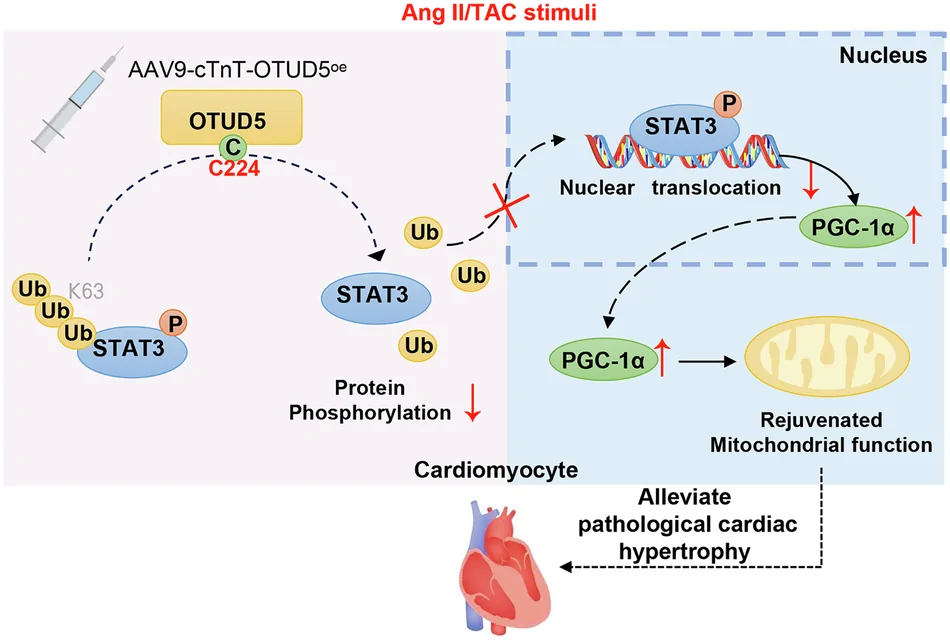

## OTUD5 enhances mitochondrial function in heart hypertrophy

Pathological cardiac hypertrophy, a major contributor to heart failure, poses important public health challenges. This study explores the role of OTUD5, a deubiquitinase, in regulating cardiac hypertrophy through a new mechanism. Researchers developed a cardiomyocyte-specific *Otud5-*knockout mouse model to investigate its function in pathological myocardial hypertrophy. They found that OTUD5 expression increased in hypertrophic cardiomyocytes, acting as a protective factor. OTUD5 deficiency exacerbated cardiac hypertrophy and dysfunction in mice, whereas its overexpression alleviated these conditions. OTUD5 achieves this by deubiquitinating STAT3 at the K283 site, inhibiting its activation and nuclear translocation, which in turn enhances PGC1α expression and mitochondrial function. These findings highlight the OTUD5–STAT3–PGC1α axis as a potential therapeutic target for heart failure, suggesting that future research can focus on developing OTUD5 modulators to improve cardiac health. "This summary was initially drafted using artificial intelligence, then revised and fact-checked by the author."

## Introduction

The acceleration of societal aging has exacerbated pathological cardiac hypertrophy and subsequent heart failure, considerably impacting public health. This condition has become an independent cause of risk for elevated cardiovascular disease incidence and mortality^1^. In response to various etiological stimuli, compensatory hypertrophy of the heart primarily manifests as an increase in the size of individual myocardial cells rather than a proliferation in cell quantity, ultimately culminating in pathological myocardial hypertrophy^2^. Consequently, to ensure the continuous contraction of myocardial cells, it is imperative that key proteins related to myocardial hypertrophy are precisely regulated to maintain contractility. Elucidating the post-translational regulatory mechanisms governing proteins, such as ubiquitination modification, will enhance our understanding of the pathogenesis underlying pathological myocardial hypertrophy.

As a member of the deubiquitinase family, OTUD5 specifically recognizes and cleaves ubiquitin chains linked to K48 and K63, thereby precisely regulating the stability and function of its substrate proteins^3^. Initially identified for its role in DNA damage repair, subsequent studies have gradually elucidated its pivotal functions in immune regulation^4^, inflammatory response^5^, and cell death^6^. OTUD5 possesses the capacity to precisely regulate cellular outcomes through distinct substrates. Notably, the importance of OTUD5 in the cardiovascular system is increasingly evident, as recent studies demonstrate its crucial role in cardiac protection during myocardial ischemia–reperfusion injury. It directly binds to glutathione peroxidase 4 (GPX4) to remove the polyubiquitin chain associated with K48 while blocking the detrimental positive feedback loop induced by 4-hydroxynonenal, thus markedly reducing myocardial injury^6^. This protective mechanism of OTUD5 has been extensively validated in ischemia/reperfusion models involving various organs such as the kidneys^7^, brain^8^, and lungs^9^. However, the role of OTUD5 in pathological myocardial hypertrophy resulting from chronic stress remains to be determined.

Based on our finding that cardiomyocyte OTUD5 is implicated in cardiac hypertrophy, we developed a cardiomyocyte-specific *Otud5*-knockout mouse model to dissect its functional role and clarify its regulatory mechanism in pathological myocardial hypertrophy. We identified a new regulatory axis centered on the deubiquitinating enzyme OTUD5, providing new and potential strategies for the treatment of pathological myocardial hypertrophy.

## Materials and methods

### Reagents

Angiotensin II (Ang II; HY-13948) and Stattic (19983-44-9) were purchased from MedChemExpress. Alexa Fluor 594-conjugated goat anti–rabbit IgG (33112ES60) and Alexa Fluor 488-conjugated goat anti–mouse IgG (33207ES60) were obtained from Shanghai Yeasen Biotechnology. Atrial natriuretic peptide (ANP) detection kits (F10062) were purchased from Xitang Biotechnology. Fluorescence staining was performed using rhodamine-conjugated phalloidin (CA1610-300T), DAPI-containing mounting medium (S2110), hematoxylin and eosin (H&E) staining kit (G1120), Picro Sirius Red staining reagent (S8060), and Masson’s trichrome staining kit (G1340) were obtained from Solarbio Life Sciences. Fluorescein isothiocyanate-conjugated wheat germ agglutinin (GTX01502) was purchased from GeneTex.

### Animal experiments

We obtained C57BL/6JGpt mice from GemPharmatech. The cardiomyocyte-specific *Otud5*-knockout (*Otud5*^fl/Y^*Myh6*-*Cre*; OTUD5CKO) mouse model was generated by crossing *Otud5* mice with *Myh6*-*Cre* transgenic mice. The *Otud5* mice (T007109) and *Myh6*-*Cre* mice (T004713) were both sourced from GemPharmatech and maintained on a C57BL/6JGpt genetic background after ten generations of backcrossing. To generate experimental animals, heterozygous *Otud5*^fl/Y^*Myh6*-*Cre*^+/−^ females were intercrossed. This breeding strategy yielded cardiomyocyte-specific *Otud5*-knockout mice (*Otud5*^fl/Y^*Myh6*-*Cre*; OTUD5CKO) and littermate control mice (*Otud5*^fl/Y^). The mice were housed in a specific pathogen-free environment and maintained at a constant temperature and humidity of 50%. Male mice aged 8 weeks (weighing approximately 21 ± 2 g) were used following approval from the Wenzhou Medical University’s Animal Ethics Committee (WYYY-AEC-YS-2025-0006). Simple randomization was performed using the sample() function in R software (v4.2.2). A fixed random seed of 20250407 was set to ensure reproducibility. Mice were allocated to each experimental group in equal ratios (e.g., 1:1 for two-group comparisons and 1:1:1:1 for four-group comparisons).

(1) The Ang II-stimulated heart failure model was established in mice by subcutaneous implantation of Alzet osmotic minipumps (Model 1004) that delivered Ang II at a dosage of 1 µg/kg/min in 100 µl saline. Following anesthesia with 2% isoflurane, the pumps were surgically placed subcutaneously near the right scapula. Blood pressure and body weight were monitored weekly, and echocardiography was performed after 4 weeks to assess the cardiac function. We randomly divided the mice into four experimental groups: *Otud5*^fl/Y^ + saline, OTUD5CKO + saline, *Otud5*^fl/Y^ + Ang II, and OTUD5CKO + Ang II groups (*n* = 6 experimental replicates). Furthermore, mice received tail vein injections of either AAV9-cTnT-NC or AAV9-cTnT-OTUD5. After injection, they were infused with Ang II (1 µg/kg/min) for 4 weeks using osmotic minipumps. We randomly divided the mice into two experimental groups: *Otud5*^fl/Y^ + AAV9-cTnT-NC + Ang II and *Otud5*^fl/Y^ + AAV9-cTnT-OTUD5^oe^ + Ang II groups (*n* = 6 experimental replicates). Stattic (a STAT3 inhibitor; 10 mg/kg) or solvent was administered orally. Echocardiography assessments occurred at 1, 3, and 4 weeks. We randomly divided the mice into three experimental groups: OTUD5CKO + AAV9-cTnT-NC + Ang II, OTUD5CKO + AAV9-cTnT- NC + Ang II + Stattic, and OTUD5CKO + AAV9-cTnT-OTUD5^oe^ +  Ang II + Stattic groups (*n* = 6 experimental replicates).

(2) The transverse aortic constriction (TAC)-induced model of chronic pressure overload heart failure was established by performing TAC via left thoracotomy on mice under 2% isoflurane anesthesia. Sham controls received identical surgical procedures without ligation. Echocardiography was performed after 4 weeks to assess cardiac remodeling. Mice were randomly divided into four groups: *Otud5*^fl/Y^ + sham, OTUD5CKO + sham, *Otud5*^fl/Y^ + TAC, and OTUD5CKO + TAC groups (*n* = 6 experimental replicates).

All experiments were approved by the Institutional Animal Care and Use Committee of Wenzhou Medical University and were performed in accordance with the relevant guidelines (GB/T 35892-2018) and the Animal Research: Reporting of In Vivo Experiments guidelines. Mice in the Ang II model were anesthetized with isoflurane inhalation (induction at 3%, maintenance at 2%). Mice in the TAC model were anesthetized with isoflurane inhalation (induction at 3%, maintenance at 2%), with oxygen supplied via a mask and mechanical ventilation. The disappearance of the pedal withdrawal reflex was used to determine whether the surgical anesthesia plane had been reached. Nonsteroidal anti-inflammatory drug meloxicam (single dose of 5 mg/kg, subcutaneous injection) was given prophylactically 30 to 60 min before the operation. Meloxicam (single dose of 5 mg/kg, subcutaneous injection) was given after the operation, once a day for a total of 3 days. Euthanasia was carried out in accordance with the American Veterinary Medical Association 2020 guidelines by first inducing deep anesthesia with 5% isoflurane and then performing cervical dislocation.

### Echocardiography

Mice were anesthetized with 3% isoflurane for induction and maintained at 2% isoflurane throughout the procedure. Subsequently, hair on the chest region was removed using electric clippers. The animals were positioned in a supine orientation on a heated imaging platform without mechanical ventilation to ensure stable body temperature. Echocardiographic examinations were conducted using a high-resolution Vevo 3100 ultrasound system for imaging small animals.

### Cell culture and transfection

NIH/3T3 cells (Cell Bank of the Chinese Academy of Sciences) were cultured in high-glucose DMEM (Gibco) containing 10% FBS (R223-00, Vazyme) in a humidified incubator at 37 °C and 5% CO_2_. HL-1 cells (Cell Bank of the Chinese Academy of Sciences) were cultured in Claycomb medium (51800C, Sigma) supplemented with norepinephrine (A0937, Sigma), FBS, and 4 mM L-glutamine (G0200, Solarbio).

Neonatal rat primary cardiomyocytes (NRCMs) were extracted from Sprague-Dawley rat pups within 72 h after birth. The lactating rats were placed in a sealed container and exposed to 5% isoflurane to induce deep anesthesia. Immediately after the anesthesia, physical methods such as decapitation or cervical dislocation were used to ensure their death. Briefly, the hearts were rapidly excised, extensively rinsed with sterile PBS to reduce residual blood, and then subjected to enzymatic digestion using trypsin (Gibco) through sequential cycles of 8-min digestion periods at 37 °C while maintaining gentle agitation. The dissociated cells were collected via centrifugation and resuspended in complete culture medium before undergoing differential adhesion to eliminate fibroblasts. The purified cardiomyocytes were cultured in DMEM, and 0.1 mM of 5-bromodeoxyuridine (HY-15910, MedChemExpress) was added to inhibit fibroblast proliferation. Cells were sustained at a temperature of 37 °C in a CO_2_ incubator set at 5%, and experiments were conducted following an incubation period of approximately 24 h after plating when spontaneous contractions became apparent.

For gene silencing, cells were transfected with *Otud5* short interfering RNA (siOTUD5; sense: 5’-GAUGCUAGAAGACAAGAAACG-3’, antisense: 3’-CUACGAUCUUCUGUUCUUUGC-5’ for mouse and 3’-ACAGCGAAGACGAAUAUGATT-5’ for rat), which was procured from GenePharma, using Lipofectamine 2000 (Thermo Fisher Scientific). For overexpression studies, Flag-OTUD5-WT (mouse and rat) and Flag-OTUD5-C224A plasmids were sourced from RiboBio. In addition, His-STAT3-WT, His-STAT3-K283R, and His-STAT3-K707R plasmids were obtained from GenScript, whereas HA-Ub, HA-K48Ub, and HA-K63Ub plasmids were acquired from Genechem. All plasmid transfections were conducted using Lipofectamine 3000 (L3000015; Thermo Fisher Scientific), following the manufacturer’s protocol.

### Western blotting

Cellular proteins were extracted using NP-40 lysis buffer (P0013F, Beyotime), and animal tissue proteins were extracted using mammalian cell lysis buffer (20D08A01, Boster). The cytosolic and nuclear proteins in cells or tissues were separated using an extraction kit (EX1470, Solarbio). The sample proteins were resolved on a 10% prepared gel (Vazyme), which was subsequently transferred onto a polyvinylidene difluoride membrane (IPVH00010, Merck Millipore). Next, the blocking step was conducted by incubating the membranes with a 10% skimmed milk solution (1172GR500, Biofroxx) prepared in TBST buffer for 1 h at room temperature. The polyvinylidene difluoride membranes were washed with TBST, and sections of the membranes were cut according to the positions of the target proteins. The membranes were then incubated with primary antibody solutions overnight at 4 °C. After incubation, the memebranes were incubated for 1 h at room temperature with the corresponding secondary antibodies. Finally, after thorough washing to remove any unbound secondary antibodies, signals on the membrane were detected using enhanced chemiluminescence reagents (NCM, P10300).

Primary antibodies, including anti-Flag (Proteintech, 20543-1-AP), anti-HA (Proteintech, 51064-2-AP), anti-His (Proteintech, 66005-1-Ig), anti-IgG (Proteintech, B900610), anti-GAPDH (Bioworld, MB001H), anti-OTUD5 (Proteintech, 21002-1-AP), anti-STAT3 (Cell Signaling Technology, 12640S), anti-phospho-Tyr705-STAT3 (Cell Signaling Technology, 9145S), anti-Lamin B (Abcam, ab133741), anti-MyHC (Abcam, ab50967), anti-ANP (Santa Cruz Biotechnology, SC-515701), anti-SORBS3 (Bai Ao Lai Bo, Z27112), and anti-SPTBN1 (Proteintech, 19722-1-AP), are listed in Supplementary Table 11.

### Co-immunoprecipitation (Co-IP)

An appropriate amount of Protein A and G-coupled agarose beads (P2055, Beyotime) was added to the cell or lysate and incubated on a rotator at low temperature to minimize nonspecific binding. After transferring the supernatant, a specific antibody was added at a dilution of 1:200, with a portion reserved as an input control. The remaining lysates were then subjected to immunoprecipitation using the beads at low temperature to stand for 5 h. The samples were then washed with PBS three to five times before proceeding with western blot analysis.

### Co-IP with liquid chromatography–tandem mass spectrometry (LC–MS/MS) analysis

HL-1 cells were transfected with either the Flag vector or Flag-OTUD5 plasmid and then stimulated with Ang II. Co-IP was performed by incubating the cell lysates with anti-Flag antibodies and protein G-Sepharose beads. The bound proteins were subsequently eluted from the beads using lysis buffer, and the resulting protein samples underwent filter-aided sample preparation for proteolytic digestion. LC–MS/MS analysis of the obtained peptides was performed by PTM Biolabs. Potential substrates were predicted from protein intensity measurements obtained from the mass spectrometry analysis.

### Quantitative real-time PCR (RT-qPCR)

Following extraction with TRIzol reagent, total cellular RNA was reverse-transcribed into complementary DNA (cDNA) using the HiScript III All-in-One RT SuperMix (Vazyme, R333-01) per the manufacturer’s instructions. An amplification system containing cDNA was then prepared using SYBR qPCR Master Mix and subjected to RT-qPCR following preset variable temperature protocols. The primer sequences used in this experiment are detailed in Supplementary Table 1.

### Human heart samples

Samples of hypertrophic myocardium were collected from patients with a clinical diagnosis of heart failure. This study was approved by the Ethics Committee of The First Affiliated Hospital of Wenzhou Medical University (KY2022-156) and was conducted in strict adherence to the principles outlined in the Declaration of Helsinki. Before collecting samples, written informed consent forms were obtained from the participants.

### Histopathological evaluation

Using paraffin-embedded sections, cardiac histology and collagen fiber analyses were evaluated by the results obtained using H&E (G1120, Solarbio) and Masson’s trichrome (G1340, Solarbio) staining, respectively. Sirius Red staining (S8060-5, Solarbio) was used to assess the pathophysiology of collagen-containing tissues. To assess the cardiomyocyte area, frozen sections were stained at room temperature using a 120 nmol/l concentration of wheat germ agglutinin (WGA) dye solution (GTX01502, GeneTex).

### Cleavage under targets and tagmentation

The thoroughly washed cell suspension was treated with a permeabilization buffer (such as buffer A with 0.01% digitonin) for 5–10 min. The cells were washed to remove excess agent while maintaining cell integrity. The primary antibody was diluted according to the manufacturer's instructions (1:1000), added to the cells, and incubated overnight at 4 °C for optimal binding. The cells were washed with washing buffer to remove unbound antibodies and minimize nonspecific signals. The premixed secondary antibody/G-Tn5 complex was added and incubated at room temperature for 1–2 h for binding. The cells were washed again to remove unbound complexes and reduce background noise. An activation buffer containing Mg^2+^ (such as tagmentation buffer) was added and incubated at 37 °C for 30 min. Mg^2+^ is essential for Tn5 activity as it cleaves DNA in target regions and ligates adapters (with sequencing primer sites) to both ends, forming “tagged fragments.” EDTA and proteinase K were then added and incubated at 55 °C for 1 h. DNA was purified using magnetic bead-based or column-based methods to eliminate impurities such as proteins and unreacted adapters. The purified DNA was amplified via PCR (using primers that bind Tn5-inserted adapters) for sufficient library yield (typically 15–20 cycles). An Agilent Bioanalyzer was used to measure library fragment size (usually 200–1000 bp), and Qubit was used to quantify concentration. High-throughput sequencing was conducted on an Illumina platform (such as NovaSeq and MiSeq).

### Immunofluorescence

The frozen heart sections were washed three times for 5 min each with PBS on a shaker and subsequently blocked with 5% BSA (A1933, Merck) for 30 min. The diluted primary antibodies, including anti-vimentin (ab8978, 1:200, Abcam), anti-α-SMA (48938, 1:200, Cell Signaling Technology), anti-CD68 (ab201973, 1:200, Abcam), and anti-OTUD5 (21002-1-AP, 1:200, Proteintech), were added to the tissue and reacted overnight in a low-temperature environment. After three additional washes with PBS, the sections were incubated with secondary antibodies at room temperature. The sections were then mounted using an antifluorescence quencher containing DAPI (0100-20, SouthernBiotech) and imaged.

### Cellular bioenergetics analysis

Mitochondrial respiratory function was evaluated via oxygen consumption rate (OCR) measurements on a Seahorse XFe96 Analyzer (Agilent Technologies). Following Ang II stimulation of HL-1 cells seeded at 5 × 10^3^ cells per well in a pre-equilibrated XF96 plate, the culture medium was replaced with an assay-specific buffer and incubated for 1 h at 37 °C without CO_2_. Using the XF Cell Mito Stress Test Kit, key parameters (basal, ATP-linked, and maximal respiration and spare respiratory capacity) were derived from real-time OCR measurements after sequential injection of oligomycin (1.5 μM), FCCP carbonyl cyanide-4-(trifluoromethoxy)phenylhydrazone (3 μM), and rotenone/antimycin A (0.5 μM). The assay was performed with technical replicates as per the manufacturer’s instructions.

### Blinding and allocation concealment

The randomization sequence was generated by an independent statistician who was not involved in animal housing, drug administration, or data collection. The investigator responsible for subcutaneous pump implantation, TAC surgery, and AAV9/Stattic administration was blinded to group assignment. The animal caretakers were also blinded to the treatment groups. Outcome assessments were performed by investigators blinded to the allocation: echocardiography images were analyzed by a trained technician who did not participate in animal handling; histological sections (H&E, WGA, Masson's trichome, and Sirius Red staining) were evaluated blindly; western blot and qPCR data were acquired and analyzed blindly. Data analysis was conducted on datasets with group labels blinded.

### Statistical analysis

Data are presented as the mean ± s.d. from at least three independent biological replicates. For in vitro studies, measurements were derived from large cell populations, justifying the assumption of normality by the central limit theorem. In vivo experiments used a sample size of *n* = 6 experimental replicates per group. The normality of all data was verified using the Shapiro–Wilk test (*P* > 0.05).

## Results

### Cardiomyocyte OTUD5 negatively regulates Ang II-induced cardiac hypertrophy and dysfunction

**Fig. 1:**
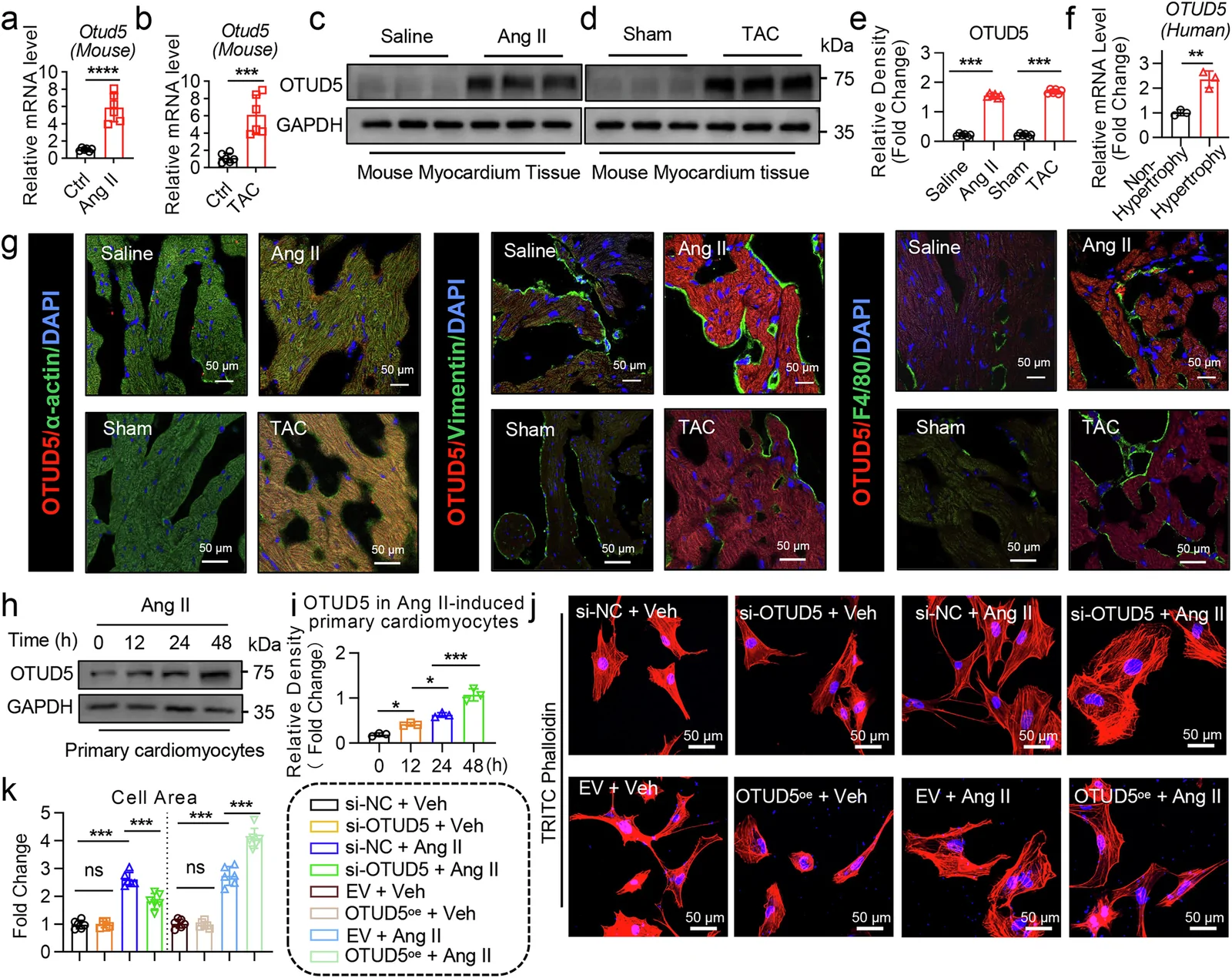
Cardiomyocyte OTUD5 negatively regulates Ang II-induced cardiac hypertrophy and dysfunction. **a**, **b** Expression of *Otud5* mRNA in Ang II (**a**)- or TAC (**b**)-induced cardiac hypertrophy in mice; *n* = 6 experimental replicates; Ctrl, control. **c–e** Expression of OTUD5 protein in Ang II-induced (**c**) or TAC-induced (**d**) cardiac hypertrophy in mice, along with the corresponding grayscale intensity analysis (**e**); *n* = 6 experimental replicates. **f** *OTUD5* mRNA levels in human hypertrophic myocardial tissues; *n* = 3 experimental replicates. **g** Immunofluorescence staining of OTUD5 (red), α-actin (green), vimentin (green), and F4/80 (green) in heart sections treated with Ang II (top) or TAC (bottom). **h**, **i** OTUD5 expression in primary cardiomyocytes (**h**) and the corresponding grayscale intensity analysis (**i**); *n* = 3 experimental replicates. **j**, **k** NRCMs were transfected with NC (negative control) or siOTUD5 or infected with empty vector (EV) or an OTUD5 overexpression plasmid, followed by Ang II (1 μM, 24 h) treatment. tetramethylrhodamine isothiocyanate (TRITC)-labeled phalloidin staining (**j**) and quantitative analysis of cell surface area (**k**); *n* = 6 experimental replicates; veh; vehicle. All data are presented as mean ± s.d. A *t*-test was used (**a**, **b**, **e**, and **f**) for comparison between two groups. For comparisons among more than two groups, ordinary one-way ANOVA was conducted (**i** and **k**); *P < 0.05, **P < 0.01, ***P < 0.001, and ****P < 0.0001; NS not significant (*P *> 0.05).

We treated mice with Ang II infusion or TAC operation to induce pathological cardiac hypertrophy and then examined the OTUD5 expression levels in mouse heart tissues. Our findings showed that both mRNA and protein levels of OTUD5 were significantly upregulated in the hearts of mice subjected to Ang II or TAC treatment, in comparison with the control groups (Fig. 1a–e). We also collected human heart samples and found that OTUD5 mRNA expression was elevated in hypertrophic myocardial tissues from patients (Fig. 1f). Immunofluorescence staining analysis further confirmed that the increased expression of OTUD5 was predominantly localized within α-actin-positive cardiomyocytes, suggesting a cardiomyocyte-specific expression pattern (Fig. 1g). In vitro experiments also showed that the level of OTUD5 protein increased significantly in a time-dependent manner in Ang II-challenged NRCMs (Fig. 1h, i). However, Ang II stimulation failed to induce OTUD5 expression in both primary cardiac fibroblasts (Supplementary Fig. 1a, b) and macrophages (Supplementary Fig. 1c, d). Functionally, we found that knockdown of OTUD5 exacerbated the Ang II-induced enlargement of NRCM cell area, whereas overexpression of OTUD5 mitigated this hypertrophic phenotype (Fig. 1j, k). Together, our findings indicate that the expression of OTUD5 in cardiomyocytes is considerably upregulated, possibly as a protective factor, during Ang II- or TAC-induced pathological cardiac hypertrophy.

### Cardiomyocyte-specific OTUD5 deficiency amplifies Ang II- and TAC-induced cardiac hypertrophy and dysfunction

To elucidate the role of cardiomyocyte OTUD5 in cardiac hypertrophy, we crossed *Otud5*^fl/Y^ mice with *Myh6*-*Cre* mice to generate cardiomyocyte-OTUD5CKO (Supplementary Table 2 and Supplementary Fig. 2a–d). Myocardial hypertrophy was induced through subcutaneous injection of Ang II (1 µg/kg/min) via a permeable micropump for 4 weeks. During the modeling period, body weight in each group steadily increased with age, and no significant differences were observed among four groups (Supplementary Fig. 3a). After Ang II treatment, systolic blood pressure did not significantly differ between OTUD5CKO and *Otud5*^fl/Y^ mice (Supplementary Fig. 3b), indicating that OTUD5 deficiency in cardiomyocytes did not impact systemic blood pressure.

Notably, upon Ang II administration, left ventricular hypertrophy became more pronounced in the OTUD5CKO group than in the controls. This was evidenced by increased anterior wall thickness and ventricular septal thickness at end-diastole, along with reduced ejection fraction and fractional shortening values (Supplementary Fig. 4a–c and Supplementary Table 3). Additionally, there were significant increases in both the heart weight-to-body weight ratio (HW/BW) and heart weight-to-tibial length ratio (HW/TL) in Ang II-infused OTUD5CKO mice when compared to their counterparts (Supplementary Table 3). We also noted that cardiac volume markedly increased in OTUD5CKO mice following Ang II induction relative to that in the *Otud5*^fl/Y^ group (Supplementary Fig. 4d). Histological analyses via H&E and WGA staining showed that OTUD5CKO exacerbated Ang II-induced enlargement of the cardiac cross-sectional area in mice (Supplementary Fig. 4e–g). Subsequently, Masson's trichrome and Sirius Red staining analyses showed that Ang II induced more serious fibrosis in OTUD5CKO mouse heart than in that of the *Otud5*^fl/Y^ group (Supplementary Fig. 4h–k). Moreover, we observed that OTUD5 deficiency aggravated MyHC and ANP overexpression in Ang II-challenged mouse heart tissues (Supplementary Fig. 4l,m), accompanied by a further increased serum ANP level (Supplementary Fig. 4n). These results show that cardiomyocyte-specific deletion of OTUD5 exacerbates Ang II-induced cardiac hypertrophy, remodeling, and dysfunction in mice.

**Fig. 2:**
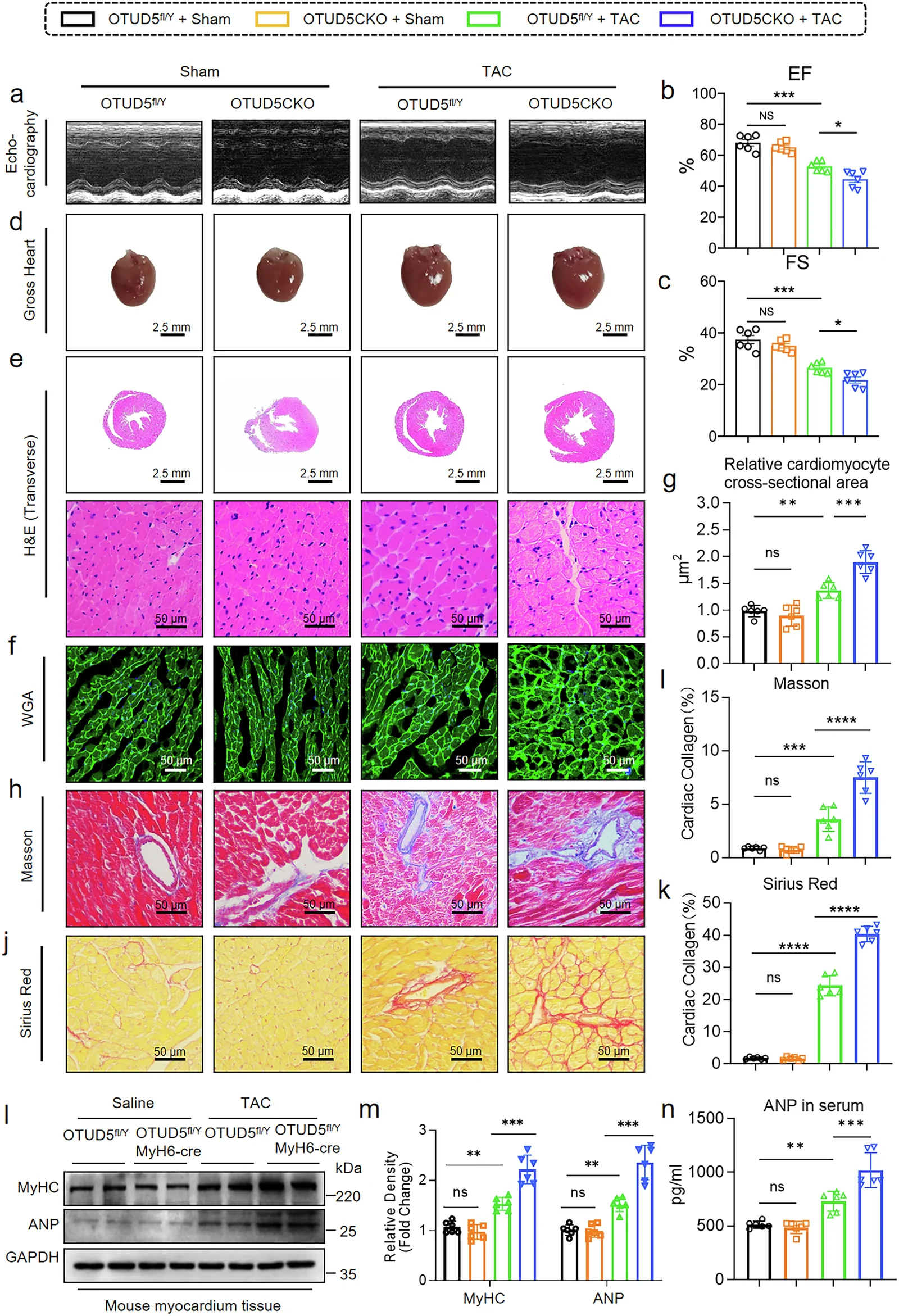
Cardiomyocyte-specific OTUD5 deficiency amplifies Ang II- and TAC-induced cardiac hypertrophy and dysfunction. ** a**–**c** OTUD5CKO and *Otud5*^fl/Y^ mice underwent either TAC or sham surgery for 4 weeks. Representative M-mode echocardiographic images of mice (**a**), ejection fraction (EF, **b**), and fractional shortening (FS, **c**) were evaluated in mice using echocardiography. **d** Representative gross heart images. **e** Representative H&E-stained heart sections. **f**, **g** Representative WGA-stained sections (**f**) and quantitative analysis of cardiomyocyte cross-sectional area (**g**). **h**, **i** Representative Masson’s trichrome-stained sections (**h**) and quantitative analysis of fibrotic area (**i**). **j**, **k** Representative Sirius Red-stained sections (**j**) and quantitative analysis of fibrotic area (**k**). **l**, **m** Protein levels of MyHC and ANP (**l**) in mouse heart tissues and quantitative analysis (**m**). **n** Serum ANP levels in mice. All data are presented as mean ± s.d. For comparisons among more than two groups, ordinary one-way ANOVA was conducted; *P < 0.05, **P < 0.01, ***P < 0.001, and ****P < 0.0001; *n* = 6 experimental replicates.

We evaluated the impact of OTUD5 in pressure overload-induced cardiac hypertrophy. We conducted a 4-week TAC operation on OTUD5CKO mice and their littermate controls, and we observed that knocking out *Otud5* in cardiomyocytes greatly exacerbated TAC-induced cardiac dysfunction (Fig. 2a–c and Supplementary Table 4). This was accompanied by an exacerbated myocardial hypertrophy in TAC-challenged OTUD5CKO mice (Fig. 2d–g and Supplementary Table 4). Furthermore, TAC operation induced more myocardial fibrosis (Fig. 2h–k) and higher MyHC and ANP levels (Fig. 2l,m) in OTUD5CKO mouse heart tissues. A similar trend was observed in serum ANP levels among the four groups (Fig. 2n). These results validate that the deficiency of cardiomyocyte OTUD5 also exacerbates TAC-induced cardiac remodeling and dysfunction.

We then examined whether cardiomyocyte-specific OTUD5 overexpression protects the heart from Ang II infusion. We administered wide-type mice with AAV9-cTnT-OTUD5^oe^ via tail vein injection, using AAV9-cTnT-NC as a control. Echocardiographic assessments were conducted at baseline time point (week 0), week 3, and week 4 after Ang II infusion (Supplementary Fig. 5a). Cardiac function analysis indicated that AAV9-cTnT-OTUD5^oe^ treatment significantly prevented Ang II-stimulated heart dysfunction, as evidenced by increased ejection fraction and fractional shortening values compared with the control group. Time-course analysis showed that the therapeutic effects of OTUD5 manifested in a time-dependent manner, emerging initially after three weeks of intervention and reaching statistical significance at week 4 (Supplementary Fig. 5b–d and Supplementary Tables 5–7). Histological analyses demonstrated that cardiomyocyte OTUD5 overexpression effectively mitigated Ang II-induced myocardial hypertrophy and fibrosis in mice (Supplementary Fig. 5e–h), along with reduced serum ANP level (Supplementary Fig. 5i). Furthermore, Masson’s trichrome staining (Supplementary Fig. 5j–k) and Sirius Red staining (Supplementary Fig. 5l–m) showed significantly reduced fibrosis in Ang II-challenged mice overexpressing OTUD5. Our study demonstrates that cardiomyocyte-specific overexpression of OTUD5 effectively alleviates Ang II-induced cardiac remodeling and dysfunction.

### OTUD5 binds to STAT3 to deubiquitinate it at the K283 site

**Fig. 3:**
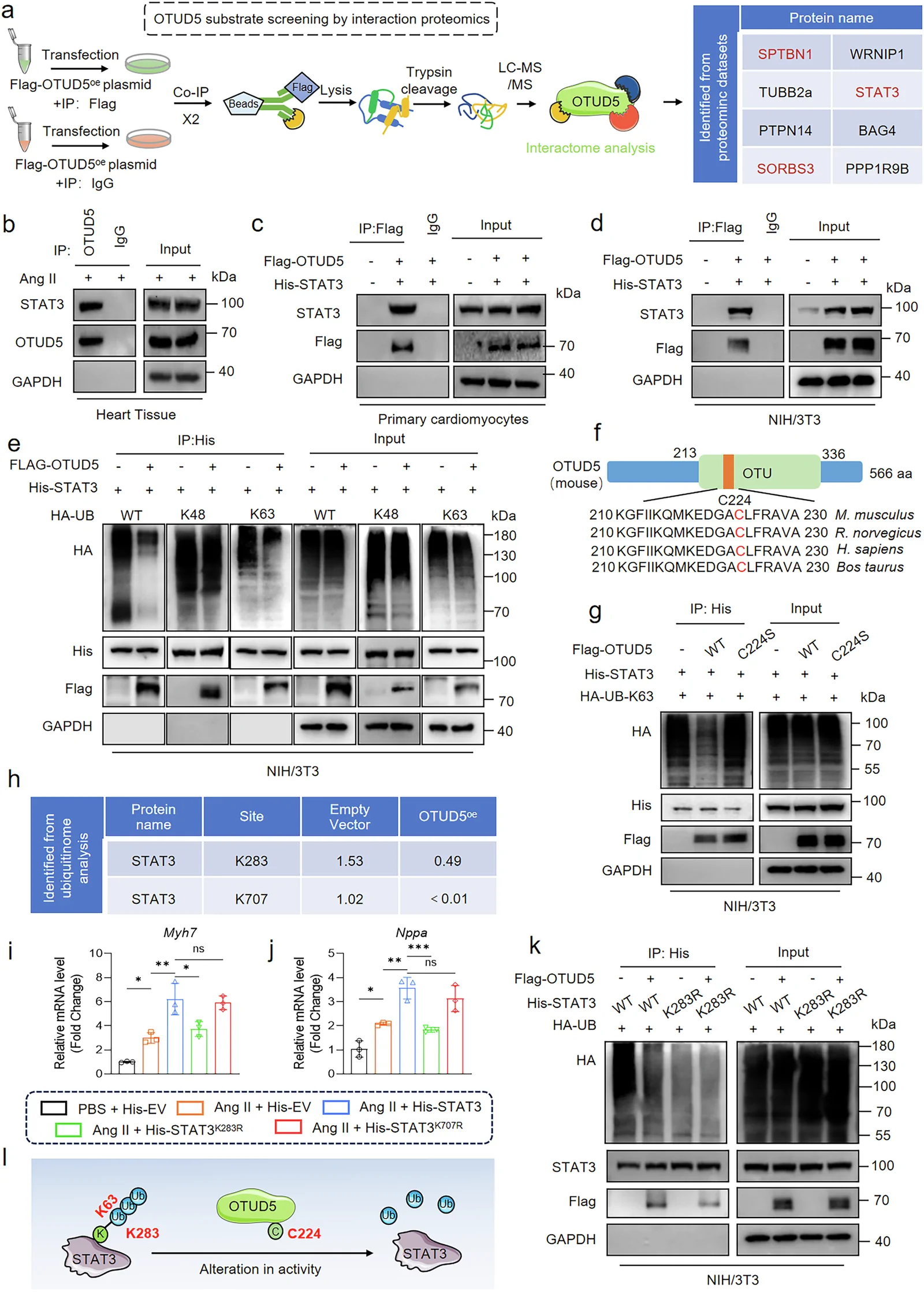
OTUD5 binds to STAT3 to deubiquitinate it at the K283 site. **a** Schematic illustration of co-IP coupled with LC–MS/MS for OTUD5 substrate screening. HL-1 cells were transfected with the Flag-OTUD5 plasmid, followed by Ang II stimulation (1 μM, 24 h). Anti-Flag and protein G-Sepharose beads were added to cell lysates for co-IP. Bound proteins were extracted, digested into peptides, and analyzed by LC–MS/MS. **b**–**d** Co-IP of OTUD5 and STAT3 in heart tissue lysates (**b**), HL-1 cells (**c**), and NIH/3T3 cells (**d**). **e** His-STAT3, HA-Ub, or its mutants (HA-K48-only or HA-K63-only) were transfected into NIH/3T3 cells with or without Flag-OTUD5, followed by MG132 treatment (10 µM). His-tagged proteins were pulled down for western blot analysis of HA, His, and Flag to determine the ubiquitination pattern regulated by OTUD5; WT, wild type. **f** Schematic of OTUD5 domain architecture and catalytic site (C224). **g** NIH/3T3 cells were transfected with Flag-OTUD5 (WT or C224S mutant), His-STAT3, and HA-Ub, followed by MG132 treatment. Ubiquitinated STAT3 was enriched by a His-tag pull-down assay and detected by western blotting for HA, His, and Flag to determine whether the C224 residue is required by OTUD5 for STAT3 deubiquitination. **h** Screening of ubiquitinated lysine residues on STAT3 regulated by OTUD5 using Co-IP-based ubiquitinated peptide enrichment analysis. **i**, **j** Effects of STAT3 site mutations on *Myh7* (**i**) and *Nppa* (**j**) mRNA levels in HL-1 cells (*n* = 3). **k** NIH/3T3 cells were transfected with His-STAT3 (WT, K283R mutant, and K707R mutant), Flag-OTUD5, and HA-Ub, followed by MG132 treatment. Ubiquitinated STAT3 was enriched by His-tag pull-down assay and detected by western blotting for HA, His, and Flag to identify the ubiquitinated lysine residues on STAT3 regulated by OTUD5. **l** Schematic of the OTUD5-mediated deubiquitination of STAT3. All data are presented as mean ± s.d. For comparisons among more than two groups, ordinary one-way ANOVA was conducted; *P < 0.05, **P < 0.01, and ***P < 0.001; *n* = 3 experimental replicates.

To identify the deubiquitination substrates of OTUD5 in cardiomyocytes, we overexpressed Flag-OTUD5 in Ang II-challenged HL-1 cells and conducted Co-IP combined with LC–MS/MS analysis, which revealed multiple proteins that physically interact with OTUD5 (Fig. 3a). Among these proteins, STAT3 (ref. ^10^), SPTBN1 (ref. ^11^), and SORBS3 (ref. ^12^) have been reported to directly participate in Ang II- and TAC-induced cardiac hypertrophy. The Co-IP assays confirmed an endogenous interaction between OTUD5 and STAT3 in Ang II-stimulated cardiac tissue, whereas OTUD5 did not directly bind to SPTBN1 and SORBS3 (Fig. 3b and Supplementary Fig. 6). These results indicate that STAT3 which is involved in cardiac hypertrophy may be the substrate of OTUD5. We also co-transfected Flag-OTUD5 and His-STAT3 plasmids into HL-1 and NIH/3T3 cells, demonstrating their exogenous association (Fig. 3c,d). Further, we observed that overexpression of OTUD5 considerably reduced the level of STAT3 ubiquitination, especially through a K63-linked manner, without affecting K48-linked ubiquitination, in NIH/3T3 cells (Fig. 3e). It is known that K48-linked ubiquitination modification mainly regulates protein stability, whereas K63 linkage preferentially regulates protein activity and function^13^. Consistently, we confirmed that OTUD5 did not affect the protein level of STAT3 in cardiomyocytes (Supplementary Fig. 7).

Sequence analysis showed that the deubiquitinating active site, located at the cysteine residue C224 of OTUD5, is highly conserved across species (Fig. 3f). The inactivated mutant C224S (Cys→Ser) completely abolished the capacity of OTUD5 to remove ubiquitin chains from STAT3 (Fig. 3g). Subsequently, we identified K283 and K707 as potential deubiquitination sites on STAT3 using a ubiquitination peptide enrichment assay (Fig. 3h). Functional assays demonstrated that the K283R mutation in STAT3 significantly attenuated Ang II-induced hypertrophic gene expression in HL-1 cells, whereas the K707R mutation did not, indicating that K283 deubiquitination negatively regulated hypertrophic gene transcription (Fig. 3i,j). Notably, the STAT3 K283R variant exhibited markedly reduced ubiquitination levels compared with wild-type STAT3, and this modification was resistant to further reduction by OTUD5 (Fig. 3k), thereby establishing K283 as the primary site for OTUD5-mediated STAT3 deubiquitination. In summary, OTUD5 deubiquitinates STAT3 at K283 via its catalytic C224 residue to regulate cardiomyocyte hypertrophy (Fig. 3l).

### OTUD5 negatively regulates cardiomyocyte hypertrophy by inhibiting STAT3 activation

**Fig. 4:**
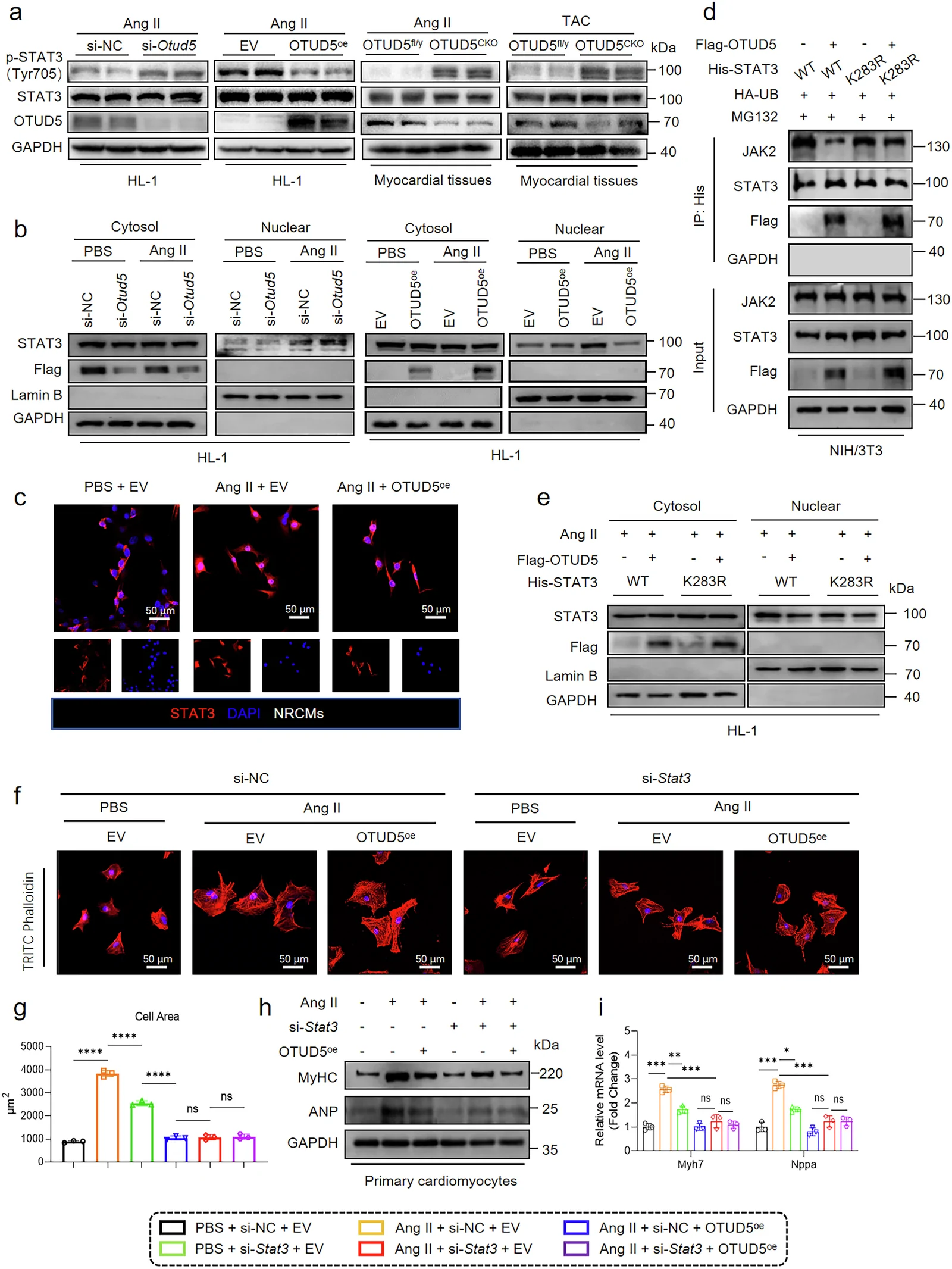
OTUD5 negatively regulates cardiomyocyte hypertrophy by inhibiting STAT3 activation. **a** Western blot analysis of p-STAT3^Y705^ and total STAT3 protein levels in HL-1 cells with OTUD5 silencing or overexpression, and in heart lysates from Ang II- or TAC-induced *Otud5*^fl/Y^ and OTUD5CKO mice. **b** Subcellular localization analysis of STAT3 by western blotting in cytoplasmic and nuclear fractions of HL-1 cells with OTUD5 silencing or overexpression. **c** Immunofluorescence microscopy showing OTUD5 regulation of STAT3 nuclear translocation in NRCMs. **d** His pull-down assay in NIH/3T3 cells overexpressing Flag-OTUD5, His-STAT3 (WT or K283R mutant), and HA-Ub under MG132 treatment, followed by western blot detection of JAK2, Flag, and STAT3 to investigate how the mutations in STAT3 affect OTUD5-mediated regulation of the STAT3–JAK2 interaction. **e** Subcellular distribution analysis of STAT3 (cytoplasmic versus nuclear fractions) by western blotting in HL-1 cells overexpressing Flag-OTUD5 and His-STAT3 (WT or K283R mutant). **f**–**i** Cells were transfected with siNC or *Stat3* siRNA (siSTAT3), and empty vector or OTUD5 plasmid, followed by PBS or Ang II stimulation. The surface area of the cardiomyocytes was assessed using TRITC-labeled phalloidin staining (**f**) accompanied by a corresponding quantitative analysis (**g**). Western blot analysis of MyHC and ANP protein levels under Ang II stimulation (**h**) and quantitative analysis (**i**). All data are presented as mean ± s.d. For comparisons among more than two groups, ordinary one-way ANOVA was conducted; *P < 0.05, **P < 0.01, ***P < 0.001, and ****P < 0.0001; *n* = 6 experimental replicates.

We then explored how OTUD5-mediated STAT3^K283^ deubiquitination regulates STAT3 activity in cardiomyocytes. Recent studies have demonstrated that Ang II substantially induces STAT3 phosphorylation at Tyr705 (Y705) in mesangial cells and cardiomyocytes^14,15^. We examined the regulatory role of OTUD5 on p-STAT3^Y^^705^ level in cardiomyocytes. Silencing OTUD5 in HL-1 cells markedly exacerbated the Ang II-induced p-STAT3 levels. Conversely, OTUD5 overexpression considerably attenuated Ang II-induced STAT3 phosphorylation. As similar phenomenon was observed in Ang II/TAC-challenged mouse myocardial tissues (Fig. 4a). STAT3 activation is contingent upon its phosphorylation and subsequent nuclear translocation^16^. Nuclear STAT3 level analysis in HL-1 cells confirmed that silencing of OTUD5 increased STAT3 nuclear translocation, whereas overexpression of OTUD5 suppressed this process (Fig. 4b). This finding was further corroborated through immunofluorescence assays performed on NRCMs (Fig. 4c). JAK2 is an upstream kinase and directly regulates the phosphorylation of STAT3 (ref. ^17^). Co-IP assays demonstrated that ectopic expression of OTUD5 markedly attenuated the interaction between JAK2 and STAT3. Crucially, substitution of STAT3 at lysine 283 with arginine completely abrogated the regulatory effect of OTUD5 on JAK2–STAT3 binding (Fig. 4d). Consistent with this observation, the K283R mutation effectively blocked OTUD5-mediated suppression of STAT3 nuclear translocation (Fig. 4e). To corroborate the functional importance of this residue, HL-1 cells were transfected with plasmids encoding either wild-type STAT3 (STAT3^WT^) or the STAT3^K283R^ mutant. Under Ang II stimulation, cells expressing STAT3^K283R^ exhibited notably diminished phosphorylation at Y705 compared with those expressing STAT3^WT^, whereas total STAT3 levels remained unaltered. Furthermore, OTUD5 overexpression failed to exert an additional inhibitory effect on p-STAT3 levels in the context of STAT3^K283R^ overexpression (Supplementary Fig. 8). Finally, to determine whether STAT3 mediates the protective effects of OTUD5, loss-of-function assays revealed that silencing STAT3 not only mitigated Ang II-induced hypertrophy but also negated the protective phenotype conferred by OTUD5 overexpression in challenged cardiomyocytes (Fig. 4f,g). Similar results were observed at both the protein and mRNA levels of MyHC and ANP (Fig. 4h,i). These results confirm that OTUD5-mediated STAT3 deubiquitination at K283 inhibits STAT3 activation by preventing STAT3–JAK2 interaction, finally protecting cardiomyocytes from pathological hypertrophy.

### The OTUD5–STAT3 axis promotes cardiac hypertrophy by regulating PGC1α-mediated mitochondrial dysfunction

**Fig. 5:**
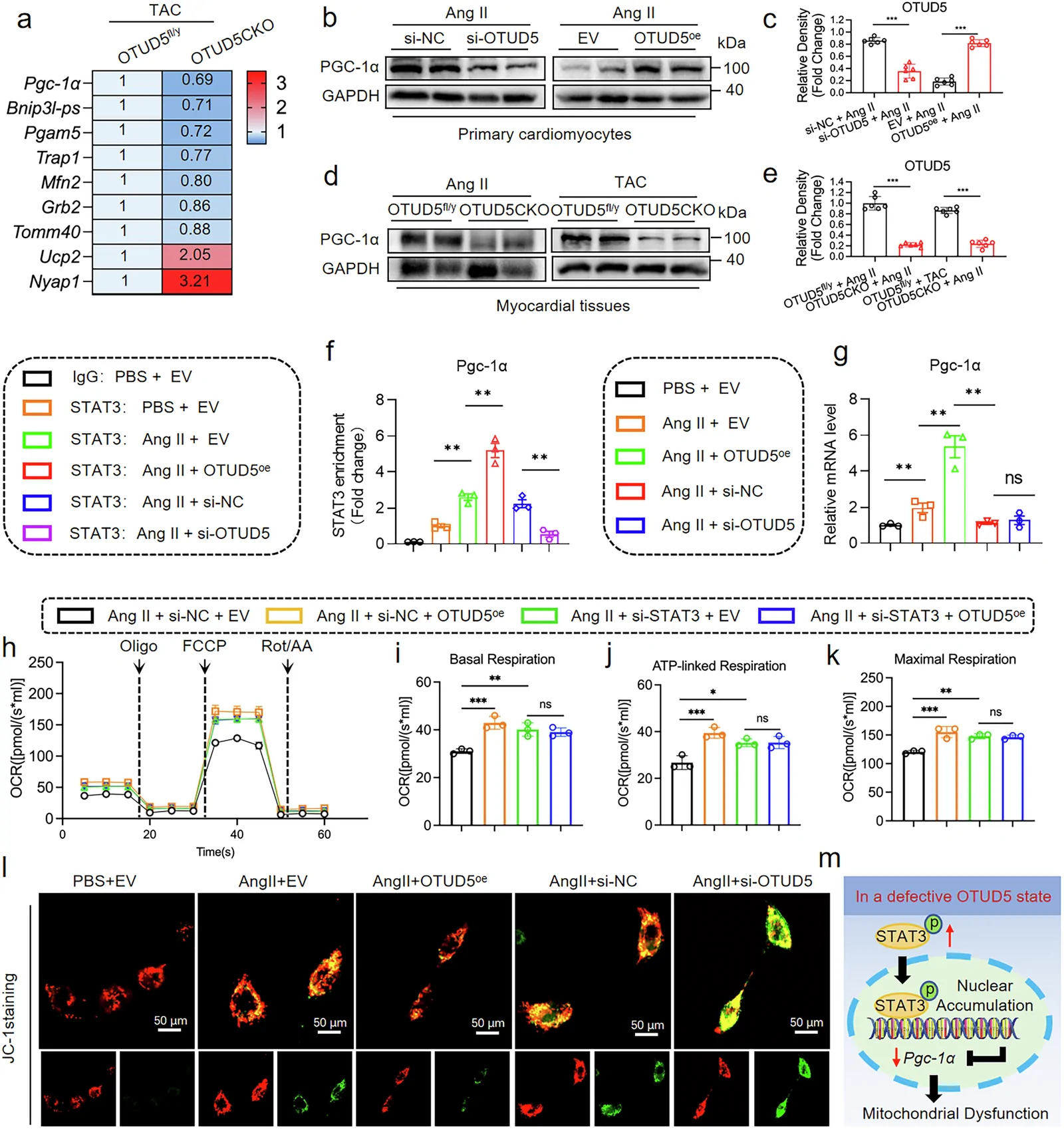
The OTUD5–STAT3 axis promotes cardiac hypertrophy by regulating PGC1α-mediated mitochondrial dysfunction. **a** Heatmap showing expression patterns of mitochondrial dynamics and biogenesis-related genes from RNA sequencing data in heart tissues of TAC-induced *Otud5*^fl/Y^ and OTUD5CKO mice. **b**–**e** Western blot analysis of PGC1α protein levels in HL-1 cells with OTUD5 silencing or overexpression (**b**) and in heart lysates from Ang II- or TAC-induced *Otud5*^fl/Y^ and OTUD5CKO mice (**d**) and their quantitative analyses, respectively (**c** and **e**); *n* = 6 experimental replicates. **f** Effects of OTUD5 overexpression or STAT3 silencing on STAT3 binding to the *Pgc1α* gene, as measured by CUT&Tag; *n* = 3 experimental replicates. **g** *Pgc1α* mRNA expression levels in HL-1 cells; *n* = 3 experimental replicates. **h**–**k** Mitochondrial respiration measurements in HL-1 cells after transfection with OTUD5 plasmid or siSTAT3, including real-time monitoring of mitochondrial oxidative capacity (**h**), basal respiration rate (**i**), ATP production-coupled respiration (**j**), and maximal respiratory capacity (**k**); *n* = 3 experimental replicates; oligo, oligomycin; FCCP, carbonyl cyanide-4-(trifluoromethoxy)phenylhydrazone; Rot/AA, rotenone/antimycin A. **l** JC-1 staining of mitochondrial membrane potential in HL-1 cells transfected with empty vector or OTUD5 plasmid, or siNC or siOTUD5, followed by PBS or Ang II stimulation. **m** Schematic representation of the mechanism by which OTUD5 regulates STAT3 nuclear activity to modulate PGC1α transcription. All data are presented as mean ± s.d. A *t*-test was used (**c** and **e**) for comparison between two groups. For comparisons among more than two groups, ordinary one-way ANOVA was conducted (**f**,** g**, and **i**–**k**); *P < 0.05, **P < 0.01, ***P < 0.001, and ****P < 0.0001.

Determining the functional impact of the OTUD5–STAT3 axis in cardiomyocytes necessitated a further investigation into the transcriptional and metabolic effects downstream of this pathway. We explored how the OTUD5–STAT3 axis regulates hypertrophic response in cardiomyocytes. As STAT3 is a transcription factor, we investigated the gene transcriptional profiles of TAC-challenged mouse heart with or without *Otud5* knockout. Notably, the OTUD5CKO mouse heart showed a notable downregulation of PGC1α, a master regulator of mitochondrial biogenesis and energy metabolism (Fig. 5a). It has been reported that dysregulation of PGC1α directly leads to mitochondrial dysfunction and energy metabolic deficits in cardiomyocytes^10^. Accordingly, we hypothesized that the OTUD5–STAT3 axis may drive mitochondrial dysfunction through regulation of PGC1α. As shown in Fig. 5b,c, silencing OTUD5 in Ang II-challenged HL-1 cells significantly reduced PGC1α expression, whereas overexpression of OTUD5 markedly upregulated it. Similarly, PGC1α protein levels were significantly diminished in myocardial tissues of OTUD5CKO mice challenged with TAC or Ang II (Fig. 5d,e). The DNA-binding activity of STAT3 with *Pgc1α* gene assessed using CUT&Tag assay validate that OTUD5 positively regulates *Pgc1α* gene transcription via STAT3 (Fig. 5f). RT-qPCR assay confirmed this result in Ang II-challenged HL-1 cells (Fig. 5g).

To validate that OTUD5 protects mitochondrial function through limiting STAT3, we transfected HL-1 cells with plasmid overexpressing OTUD5 and siRNA targeting *stat3*. The OCR results indicated that overexpression of OTUD5 significantly mitigated mitochondrial oxidative phosphorylation (OXPHOS) induced by Ang II (Fig. 5h), as evidenced by enhanced induction of basal respiration (Fig. 5i), ATP production (Fig. 5j) or maximal respiration (Fig. 5k). Importantly, silencing of STAT3 completely abolished these protective effects of OTUD5. JC-1 staining confirmed that exposure to Ang II led to important mitochondrial depolarization in OTUD5-silenced cells, whereas overexpression of OTUD5 markedly attenuated this phenotype (Fig. 5l). Collectively, these findings reveal that the OTUD5 prevents mitochondrial dysfunction by inhibiting STAT3 activity and thereby upregulating PGC1α expression in cardiomyocytes (Fig. 5m).

### OTUD5 protects against Ang II-induced cardiac dysfunction through STAT3 in vivo

**Fig. 6:**
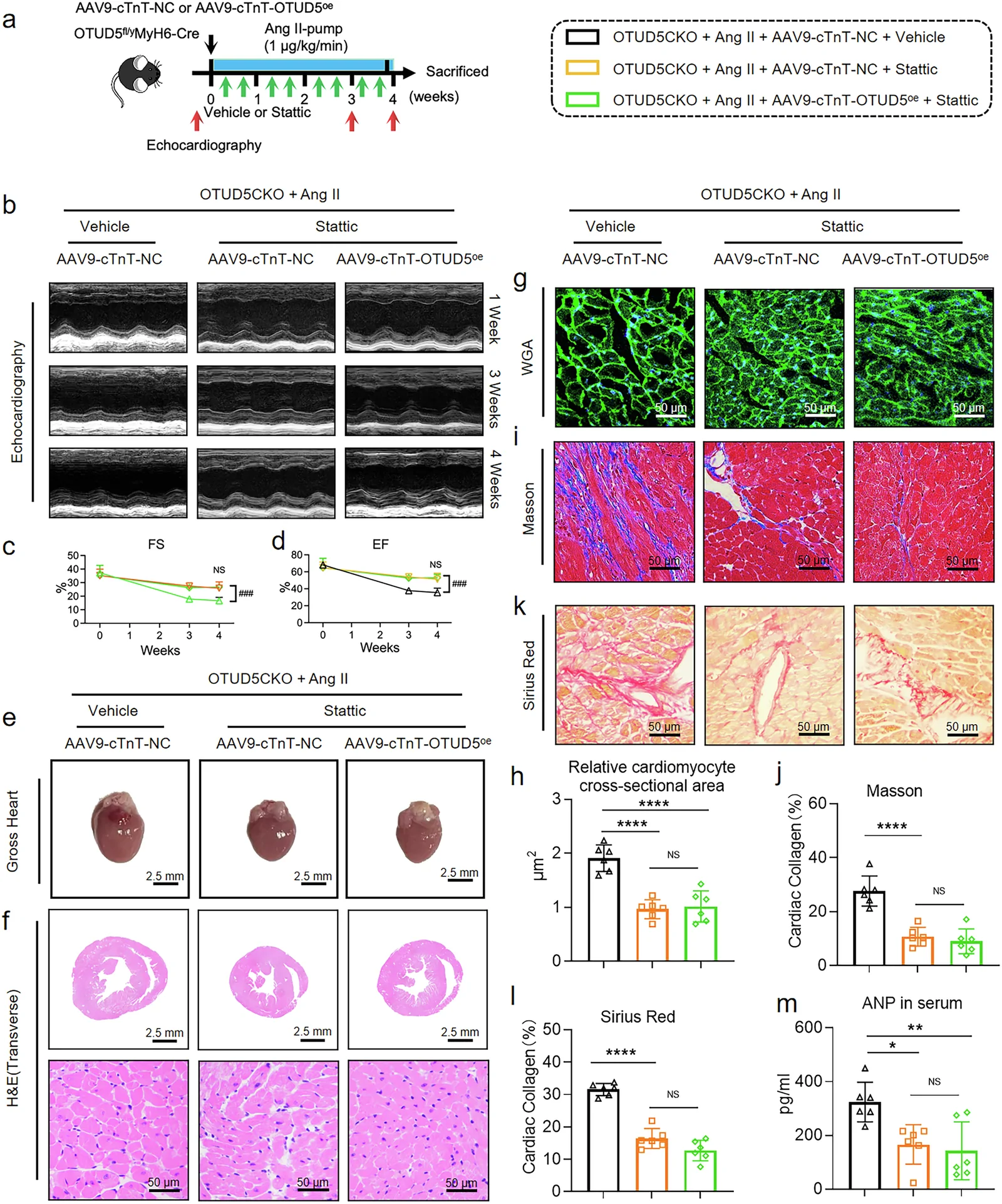
OTUD5 protects against Ang II-induced cardiac dysfunction through STAT3 in vivo. **a** OTUD5CKO mice received tail vein injections of either AAV9-cTnT-NC or AAV9-cTnT-OTUD5, followed by subcutaneous implantation of osmotic minipumps delivering either saline or Ang II (1 µg/kg/min) for 4 weeks. During the 4-week treatment period, mice were orally administered either the STAT3-specific inhibitor Stattic (10 mg/kg) or vehicle control via gavage. Cardiac function assessments were performed at weeks 1, 3, and 4. Schematic illustration of the experimental model establishment (**a**). **b**–**d** Representative M-mode echocardiography of mice (**b**), ejection fraction (EF, **c**), and fractional shortening (FS, **d**) were evaluated in mice through echocardiography. **e** Representative gross heart images. **f** Representative H&E-stained heart sections. **g**, **h** Representative WGA-stained sections (**g**) and quantitative analysis of cardiomyocyte cross-sectional area (**h**). **i**,** j** Representative Masson’s trichrome-stained sections (**i**) and quantitative analysis of fibrotic area (**j**). **k**, **l** Representative Sirius Red-stained sections (**k**) and quantitative analysis of fibrotic area (**l**). **m** Serum ANP levels in mice. All data are presented as mean ± s.d. For comparisons among more than two groups, ordinary one-way ANOVA was conducted; *P < 0.05, **P < 0.01, ***P < 0.001, and ****P < 0.0001; *n* = 6 experimental replicates.

To confirm the role of STAT3 as a functional substrate of OTUD5 in the mouse heart, AAV9-cTnT-OTUD5^oe^ was administered to OTUD5CKO mice via tail vein injection, followed by oral delivery of the STAT3-specific inhibitor Stattic. These mice were then infused with Ang II for 4 weeks (Fig. 6a). Echocardiographic analysis demonstrated that either OTUD5 ‌restoration or STAT3 inhibition significantly improved cardiac function in Ang II-infused OTUD5CKO mice (Fig. 6b–d and Supplementary Tables 8–10). However, overexpression of OTUD5 in mice, following STAT3 inhibition, did not result in a further improvement in Ang II-induced cardiac dysfunction. Histological assessments, including measurements of cardiac size (Fig. 6e), H&E staining (Fig. 6f and Supplementary Fig. 9), WGA staining (Fig. 6g–h), Masson's trichrome staining (Fig. 6i,j), and Sirius Red staining (Fig. 6k,l), revealed that Sttatic effectively mitigated myocardial hypertrophy and fibrosis stimulated by Ang II in OTUD5CKO mice. Nevertheless, OTUD5 overexpression did not provide additional benefits in alleviating Ang II-induced myocardial hypertrophy and fibrosis in Stattic-treated mice. Furthermore, serum analysis of ANP levels corroborated these findings (Fig. 6m). In conclusion, our results suggest that OTUD5 alleviates Ang II-stimulated cardiac remodeling and dysfunction by inactivating STAT3.

## Discussion

In this research, we discovered that the expression of OTUD5 is considerably upregulated in hypertrophic myocardial tissue. Cardiomyocyte-specific knockout of OTUD5 exacerbates Ang II- and TAC-stimulated hypertrophy, as well as cardiac dysfunction. Conversely, AAV9-mediated cardiomyocyte-specific overexpression of OTUD5 markedly alleviates Ang II-stimulated pathological myocardial hypertrophy. Mechanistically, STAT3 has been identified as a target of OTUD5 in hypertrophic cardiomyocytes. OTUD5 modulates the activity of STAT3 by removing the K63-linked polyubiquitin chain on STAT3 at K283 through its catalytic residue C224. This deubiquitination event facilitated by OTUD5 inhibits both the phosphorylation and nuclear translocation of STAT3, thereby relieving the suppression of PGC1α gene transcription mediated by STAT3 and ultimately enhancing mitochondrial function in cardiomyocytes. Furthermore, we validated the pivotal role of the OTUD5–STAT3 axis in Ang II-stimulated pathological cardiac hypertrophy in mice.

STAT3 plays a pivotal part in regulating cell cycle progression and inhibiting apoptosis, making it closely associated with tumor advancement and poor prognostic outcomes across various cancer types^18^. In addition, STAT3 is integral to the pathophysiology of autoimmune and inflammatory diseases such as rheumatoid arthritis and inflammatory bowel disease^19^. Recently, there has been a growing interest in the participation of STAT3 and its signaling pathways in cardiovascular pathophysiology, particularly its important contribution to pathological cardiac hypertrophy^20–23^, thereby rendering it an appealing target for therapeutic intervention. However, the development of highly effective and selective STAT3 inhibitors poses considerable challenges due to the absence of a conventional active site and its high homology with other members of the STAT family. The initial molecular probes, including polypeptides, pseudopeptides, and oligonucleotides, have shown limitations such as poor stability, low affinity, and restricted cell permeability^24,25^. Small molecules aimed at targeting the SH2 domain (e.g., TTI-101 (ref. ^26^)) provide direct action but frequently encounter issues related to selectivity and pharmacokinetics. Consequently, the focus of research has shifted toward modulating the regulatory mechanisms of STAT3. Studies have demonstrated that the stability and activity of STAT3 are intricately regulated by ubiquitination. Several deubiquitinating enzymes have been identified as key regulators of K48- or K63-linked ubiquitination of STAT3. For instance, USP28 (ref. ^27^), OTUB1 (ref. ^28^), and YOD1 (ref. ^21^) remove K48-linked ubiquitination that affects the stability of STAT3, whereas CYLD^29^, TRAF6 (ref. ^30^), OTUD1 (ref. ^31^), and USP20 (ref. ^32^) deubiquitinate STAT3 in a K63-linked manner to influence STAT3 phosphorylation or nuclear translocation. Notably, our study elucidates that STAT3 is a critical substrate of OTUD5 in cardiomyocytes, with this regulatory axis considerably contributing to pathological cardiac hypertrophy. Unlike these related enzymes, OTUD5 uniquely targets STAT3 at K283 within its DNA-binding domain rather than at degradation-associated sites such as K97 or K180. Furthermore, OTUD5 exhibits selective removal of K63-linked ubiquitin chains to modulate STAT3 transcriptional activity without compromising protein stability, contrasting with the K48-linkage specificity observed for YOD1 and OTUD1 that promotes STAT3 stabilization. Functionally, OTUD5 alleviates STAT3-mediated repression of PGC1α and enhances mitochondrial bioenergetics, establishing a regulatory axis not previously reported for other deubiquitinating enzymes. Furthermore, we highlight the cardiomyocyte-specific upregulation of OTUD5 under hypertrophic stress, a pattern not seen in fibroblasts or macrophages, underscoring its cell-autonomous protective role. Collectively, these distinctions emphasize that OTUD5 operates via a previously unrecognized mechanistic paradigm, thereby reinforcing the significance of our findings. This finding provides fresh insights into developing precise modulators for STAT3 activity.

One of the key innovations of this study lies in elucidating the unique regulatory role of ubiquitination at the K283 site of STAT3. Unlike other known post-translational modification sites on STAT3, including K49 dimethylation^33^, K97 and K180 ubiquitination^21,34^, and K140 methylation^35^, which are primarily associated with proteasomal degradation, K283 is uniquely situated within the DNA-binding domain of STAT3. This distinctive localization suggests potentially new functions beyond conventional protein stability regulation. Our findings reveal a notable correlation between K283 ubiquitination and the JAK2–STAT3 interaction, as well as the effect of JAK2 on STAT3 Y705 phosphorylation. From a structural biology perspective, there is no direct physical interaction interface between the STAT3 K283 site and JAK2 interaction. The phosphorylation mediated by JAK2 at STAT3 Y705 occurs indirectly through the binding of STAT3’s SH2 domain to phosphorylated tyrosine motifs. Interestingly, K283 resides in the DNA-binding domain (β-barrel core) of STAT3 and is spatially distant from the SH2 domain responsible for receiving JAK2 signals^36^. Therefore, we propose that K283 ubiquitination modification likely modulates the JAK2–STAT3 interaction via an allosteric mechanism. On the one hand, K283 ubiquitination may induce a conformational change in STAT3 that remotely influences the binding efficiency of its SH2 domain with JAK2. On the other hand, the ubiquitin chain attached to K283 could function as a molecular “handle”, specifically recruiting scaffold proteins containing ubiquitin-binding domains, indirectly facilitating enhanced localization of STAT3 around JAK2 signaling complexes. This discovery not only provides a novel perspective on the intricate regulatory mechanism governing STAT3 activity but also offers potential avenues for the development of innovative therapeutics targeting the JAK2–STAT3 signaling pathway.

Here we identified PGC1α as a crucial downstream effector of the OTUD5–STAT3 signaling axis in mediating mitochondrial biogenesis and cardiac function. As a master regulator of mitochondrial biogenesis and energy metabolism, PGC1α orchestrates the transcriptional activation of genes involved in oxidative phosphorylation and mitochondrial function by coactivating nuclear receptors such as peroxisome proliferator-activated receptors, estrogen-related receptors, and nuclear respiratory factors^37^. It suppresses the expression of genes contributing to mitochondrial dysfunction and metabolic impairment in cardiomyocytes^10,38^. In our study, we observed a robust positive correlation between levels of PGC1α expression and OTUD5. Accumulating evidence indicates that STAT3 can negatively regulate PGC1α expression through direct or indirect mechanisms^39,40^. These findings provide insights underlying metabolic abnormalities associated with cardiac hypertrophy and suggest that the OTUD5–STAT3–PGC1α axis may be a potential target for the treatment of heart failure. There have been no reports of small molecule agonists or activators targeting OTUD5. Currently, the strategies for enhancing the function of OTUD5 are entirely dependent on AAV-mediated gene overexpression. This method has demonstrated notable preclinical efficacy in various disease models, including diabetic nephropathy^41^, myocardial ischemia–reperfusion injury^6^, acute kidney injury models^7^, and our current findings in cardiomyopathy. In this study, we confirm that the protective effect of OTUD5 depends on its cysteine C224 active site, which can specifically remove K63-linked ubiquitin chains in STAT3 without affecting the stability of the STAT3 protein. The C224 catalytic site and the adjacent substrate-binding groove in the structure could be used in drug design to construct allosteric small-molecule agonists or molecular adhesives that enhance the endogenous activity of OTUD5 and improve pathological myocardial remodeling.

Despite these important advancements, several areas warrant further exploration. First, our current assays do not definitively distinguish K63‑linked polyubiquitination from mono‑ or multimonoubiquitination at STAT3^K283^. Future site‑specific ubiquitin stoichiometry studies will be required to clarify the precise nature and functional relevance of this modification. Although we have established STAT3 as a substrate of OTUD5, it is crucial to consider that OTUD5 may also regulate other substrate proteins implicated in cardiomyocyte hypertrophy. Second, although our study identifies PGC1α as a key downstream effector, it remains uncertain whether OTUD5 directly or indirectly regulates other mitochondrial-associated transcription factors such as ERRα, NRF1/2, and TFAM. Understanding how these factors, within the context of biogenesis and function of mitochondria, coordinate with PGC1α is very important. This limitation underscores the complexity of STAT3’s downstream regulatory network in hypertrophic cardiomyocytes. STAT3 is known to influence multiple signaling pathways beyond the PGC1α axis. As a central signaling node, STAT3 modulates diverse cellular processes and pathways. It regulates cell survival and proliferation via *Bcl2* and cyclin D1 (ref. ^42^), mediates angiogenesis through VEGF induction^43^, facilitates immune evasion via PD-L1 regulation^44^, and promotes tissue fibrosis through TGFβ1 pathway activation^45^. This multipathway coordination suggests that OTUD5-mediated regulation of STAT3 may exert broader biological effects. Future research should adopt systematic methodologies, including chromatin immunoprecipitation sequencing and transcriptomic analyses, to comprehensively delineate the downstream cascades of STAT3. Finally, advancing cardiomyocyte-specific delivery methods for OTUD5 and validating the safety profiles of OTUD5-overexpressing therapies are pivotal directions for future translational investigations.

In conclusion, our research identifies the protective role of cardiomyocyte OTUD5 in pathological myocardial hypertrophy and elucidates the underlying mechanism. We establish a comprehensive molecular cascade that connects deubiquitination, STAT3 transcriptional regulation, PGC1α expression, and mitochondrial bioenergetics. This study is anticipated to provide potential targets for the development of therapeutic interventions combating heart failure through the OTUD5–STAT3–PGC1α axis.

## Supplementary information

Supplementary Information
