# Supplementary material for: Cardiomyocyte-enriched OTUD5 alleviates pathological myocardial hypertrophy by deubiquitinating STAT3 and modulating the STAT3–PGC-1α axis
Source: Exp Mol Med. 2026 Oct 16;58(10):3579–92. doi: 10.1038/s12276-026-01870-5 (PMC13736899; doi:10.1038/s12276-026-01870-5)
Supplement: Supplementary file 1 [file 12276_2026_1870_MOESM1_ESM.pdf]

Supplementary Files

Cardiomyocyte-enriched OTUD5 alleviates pathological myocardial hypertrophy by deubiquitinating STAT3 and modulating the STAT3-PGC-1α axis

Supplementary File

Supplementary Material and Methods, 11 Supplementary Tables, 9 Supplementary Figures and Legends

Supplementary Table 1

Primer sequences for qPCR

Gene	Species	Primer sequence (5'→3')	Primer sequence (3'→ 5')
Actb	Mouse	CCGTGAAAAGATGACCCAGA	TACGACCAGAGGCATACAG
Otud5	Mouse	AAGTCTCCCTGTGTGTTTCTTGTCG	ACTCAGAAGGCAGAGGCAGAAGG
Myh7	Mouse	CAGAACACCAGCCTCATCAACCAG	TTCTCCTCTGCGTTCCTACACTCC
Nppa	Mouse	AAGAACCTGCTAGACCACCTGGAG	TGCTTCCTCAGTCTGCTCACTCAG
Pgc-1α	Mouse	GTGCCACCGCCAAACCAAGAG	TTCCTCGTGTCCTCGGCTGAG
Actb	Rat	CCTAGACTTCGAGCAAGAGA	GGAAGGAAGGCTGGAAGA
Myh7	Rat	GAGGAGAGGGCGGACATT	ACTCTTCATTCAGGCCCTTG
Nppa	Rat	GGGCTCCTTCTCCATCACC	CCTAAAACAACCTCAGCCCGT
Actb	Human	CCTGGCACCCAGCACAAT	GCCGATCCACACGGAGTACT
Otud5	Human	GTGTTAGCTACCCATCGGAAAT	ACCCTGGTTTGAATGATGG

Supplementary Table 2

Mouse gene heart-specific knockout identification primers

Primer number	Primer name	Primer sequence (5’→3’)	Product size	remark
1	JS00028-Otud5-5wt-tF1A	GTGACCACCTCACCAGTATAGGCT	WT: 327bp	Identification of Otud5 with X inheritance
	JS00028-Otud5-5wt-tR1A	GCCAGAAGCAGACAGCTTGACCT	Fl: 405bp	
2	T004713-F1	CCTGCTGTCCATTCCTTATTCCATA	WT:0bp	Identification of myh6-Cre
	T004713-R1	ATATCCCCTTGTTCCCTTTCTGC	Targeted:329bp	
3	H11-wt-tF1	CAGCAAAACCTGGCTGTGGATC	WT: 412bp	
	H11-wt-tR1	ATGAGCCACCATGTGGGTGTC		

Supplementary Table 3

Biometric and echocardiographic parameters in Ang II-challenged mouse experiment

	Saline		Ang II	
	$OTUD5^{fl/y}$ n=6 experimental replicates	OTUD5CKO n=6 experimental replicates	$OTUD5^{fl/y}$ n=6 experimental replicates	OTUD5CKO n=6 experimental replicates
HW/BW, (mg/g)	6.04±0.52	$5.86±0.39^{ns}$	7.29±0.43**	$8.56±0.61^{\#\#}$
HW/TL, (mg/mm)	7.97±0.54	$7.88±1.06^{ns}$	10.19±0.61**	$11.94±1.13^{\#}$
LVID;d, (mm)	3.40±0.54	$3.45±0.35^{ns}$	$3.84±0.38^{ns}$	$4.57±0.92^{\#\#}$
LVID;s, (mm)	2.09±0.36	$2.21±0.28^{ns}$	2.83±0.31*	$3.89±0.84^{\#\#\#\#}$
LVAW;d, (mm)	0.79±0.23	$0.81±0.11^{ns}$	$0.91±0.38^{ns}$	$0.99±0.20^{NS}$
LVAW;s, (mm)	1.31±0.28	$1.24±0.07^{ns}$	$1.31±0.28^{ns}$	$1.34±0.26^{NS}$
IVS;d, (mm)	0.79±0.10	$0.75±0.15^{ns}$	$0.79±0.14^{ns}$	$0.82±0.14^{NS}$
IVS;s, (mm)	1.11±0.16	$1.09±0.09^{ns}$	$1.00±0.17^{ns}$	$1.00±0.10^{NS}$

HW, heart weight; BW, body weight; TL, tibial length. LVID, left ventricular internal diameter; LVAW, left ventricular anterior wall; IVS, interventricular septum; d, diastole; s, systole. ns, represents p>0.05 vs $OTUD5^{fl/y}$ + Saline; *, p<0.05 vs $OTUD5^{fl/y}$ + Saline; **, p<0.01 vs $OTUD5^{fl/y}$ + Saline; NS, represents p>0.05 vs $OTUD5^{fl/y}$ + Ang II; #, p<0.05 vs $OTUD5^{fl/y}$ + Ang II; ##, p<0.01 vs $OTUD5^{fl/y}$ + Ang II; ####, p<0.0001 vs $OTUD5^{fl/y}$ + Ang II.

All data are presented as mean ± SD. For comparisons among more than two groups, Ordinary one-way ANOVA analysis was conducted.

Supplementary Table 4

Biometric and echocardiographic parameters in TAC-challenged mouse experiment

	Sham		TAC	
	$OTUD5^{fl/y}$ n=6 experimental replicates	OTUD5CKO n=6 experimental replicates	$OTUD5^{fl/y}$ n=6 experimental replicates	OTUD5CKO n=6 experimental replicates
HW/BW, (mg/g)	5.73±0.39	$5.59±0.32^{ns}$	6.59±0.55*	$7.63±0.65^{##}$
HW/TL, (mg/mm)	7.49±0.66	$7.47±0.46^{ns}$	9.06±0.95**	$10.51±0.88^{#}$
LVID;d, (mm)	3.71±0.37	$3.60±0.35^{ns}$	$3.52±0.29^{ns}$	$3.93±0.46^{NS}$
LVID;s, (mm)	2.33±0.28	$2.34±0.31^{ns}$	$2.59±0.26^{ns}$	$3.08±0.45^{##}$
LVAW;d, (mm)	0.81±0.04	$0.87±0.16^{ns}$	1.18±0.20**	$0.88±0.19^{NS}$
LVAW;s, (mm)	1.39±0.07	$1.41±0.20^{ns}$	$1.57±0.20^{ns}$	$1.21±0.20^{NS}$
IVS;d, (mm)	0.82±0.08	$0.74±0.11^{ns}$	1.09±0.21*	$0.91±0.20^{NS}$
IVS;s, (mm)	1.22±0.29	$1.12±0.11^{ns}$	$1.25±0.28^{ns}$	$1.03±0.15^{NS}$

HW, heart weight; BW, body weight; TL, tibial length. LVID, left ventricular internal diameter; LVAW, left ventricular anterior wall; IVS, interventricular septum; d, diastole; s, systole. ns, represents p>0.05 vs $OTUD5^{fl/y}$ + Sham; *, p<0.05 vs $OTUD5^{fl/y}$ + Sham; **, p<0.01 vs $OTUD5^{fl/y}$ + Sham; NS, represents p>0.05 vs $OTUD5^{fl/y}$ + TAC; #, p<0.05 vs $OTUD5^{fl/y}$ + TAC; ##, p<0.01 vs $OTUD5^{fl/y}$ + TAC.

All data are presented as mean ± SD. For comparisons among more than two groups, Ordinary one-way ANOVA analysis was conducted.

Supplementary Table 5

Echocardiographic parameters of Ang II -challenged and injected with AAV-OTUD5 mouse experiment at the first week

	AAV-NC + Ang II<br>$OTUD5^{fl/y}$<br>n=6 experimental replicates	AAV-OTUD5 + Ang II<br>$OTUD5^{fl/y}$<br>n=6 experimental replicates
LVID;d, (mm)	3.78±0.32	3.79 ± 0.21^{NS}
LVID;s, (mm)	2.46±0.26	2.36 ± 0.18^{NS}
LVAW;d, (mm)	0.76±0.10	0.77 ± 0.09^{NS}
LVAW;s, (mm)	1.24±0.17	1.30 ± 0.15^{NS}
IVS;d, (mm)	0.65±0.05	0.73±0.04**
IVS;s, (mm)	0.98±0.05	1.15±0.04****

LVID, left ventricular internal diameter; LVAW, left ventricular anterior wall; IVS, interventricular septum; d, diastole; s, systole. NS, represents p>0.05 vs $OTUD5^{fl/y}$ + AAV-NC + Ang II; **, p<0.01 vs $OTUD5^{fl/y}$ + AAV-NC + Ang II; **, p<0.0001 vs $OTUD5^{fl/y}$ + AAV-NC + Ang II.

All data are presented as mean ± SD. Between the two groups, t-test was used.

Supplementary Table 6

Echocardiographic parameters of Ang II -challenged and injected with AAV-OTUD5 mouse experiment at the third week

	AAV-NC + Ang II $OTUD5^{fl/y}$ n=6 experimental replicates	AAV-OTUD5 + Ang II $OTUD5^{fl/y}$ n=6 experimental replicates
LVID;d, (mm)	3.29±0.28	3.66±0.56^{NS}
LVID;s, (mm)	2.50±0.25	2.76±0.49^{NS}
LVAW;d, (mm)	0.79±0.11	0.85±0.24^{NS}
LVAW;s, (mm)	1.10±0.12	1.23±0.26^{NS}
IVS;d, (mm)	0.83±0.11	0.95±0.11^{NS}
IVS;s, (mm)	0.96±0.14	1.05±0.13^{NS}

LVID, left ventricular internal diameter; LVAW, left ventricular anterior wall; IVS, interventricular septum;d, diastole; s, systole. NS, represents $p>0.05$ vs $OTUD5^{fl/y}$ + AAV-NC + Ang II.

All data are presented as mean ± SD. Between the two groups, t-test was used.

Supplementary Table 7

Echocardiographic parameters of Ang II -challenged and injected with AAV-OTUD5 mouse experiment at the fourth week

	AAV-NC + Ang II $OTUD5^{fl/y}$ n=6 experimental replicates	AAV-OTUD5 + Ang II $OTUD5^{fl/y}$ n=6 experimental replicates
LVID;d, (mm)	3.15±0.25	3.57±0.34*
LVID;s, (mm)	2.46±0.20	$2.45±0.23^{NS}$
LVAW;d, (mm)	1.03±0.25	$0.97±0.16^{NS}$
LVAW;s, (mm)	1.30±0.25	$1.44±0.19^{NS}$
IVS;d, (mm)	1.19±0.30	$1.00±0.21^{NS}$
IVS;s, (mm)	1.22±0.26	$1.25±0.19^{NS}$

LVID, left ventricular internal diameter; LVAW, left ventricular anterior wall; IVS, interventricular septum;d, diastole; s, systole. NS, represents p>0.05 vs $OTUD5^{fl/y}$ + AAV-NC + Ang II; *, p<0.05 vs $OTUD5^{fl/y}$ + AAV-NC + Ang II.

All data are presented as mean ± SD. Between the two groups, t-test was used.

Supplementary Table 8

Echocardiographic parameters of Ang II -challenged and injected with AAV-OTUD5 and STAT3 inhibition mouse experiment at the first week

	AAV-NC + Ang II<br>OTUD5CKO<br>n=6 experimental replicates	AAV-NC + Ang II + Stattic<br>OTUD5CKO<br>n=6 experimental replicates	AAV-OTUD5 + Ang II+ Stattic<br>OTUD5CKO<br>n=6 experimental replicates
LVID;d, (mm)	3.54±0.26	4.10±0.52^{ns}	3.79±0.43^{NS}
LVID;s, (mm)	2.22±0.18	2.67±0.49^{ns}	2.47±0.51^{NS}
LVAW;d, (mm)	0.65±0.10	0.76±0.09^{ns}	0.72±0.08^{NS}
LVAW;s, (mm)	1.19±0.08	1.34±0.13*	1.27±0.10^{NS}
IVS;d, (mm)	0.73±0.13	0.78±0.13^{ns}	0.79±0.12^{NS}
IVS;s, (mm)	1.02±0.15	1.16±0.11^{ns}	1.07±0.14^{NS}

LVID, left ventricular internal diameter; LVAW, left ventricular anterior wall; IVS, interventricular septum; d, diastole; s, systole. ns, represents p>0.05 vs OTUD5$^{fl/y}$ + AAV-NC + Ang II; *, p<0.05 vs OTUD5$^{fl/y}$ + AAV-NC + Ang II; NS, represents p>0.05 vs OTUD5$^{fl/y}$ + AAV-NC + Ang II + Stattic.

All data are presented as mean ± SD. For comparisons among more than two groups, Ordinary one-way ANOVA analysis was conducted.

Supplementary Table 9

Echocardiographic parameters of Ang II -challenged and injected with AAV-OTUD5 and STAT3 inhibition mouse experiment at the third week

	AAV-NC + Ang II OTUD5CKO n=6 experimental replicates	AAV-NC + Ang II + Stattic OTUD5CKO n=6 experimental replicates	AAV-OTUD5 + Ang II+ Stattic OTUD5CKO n=6 experimental replicates
LVID;d, (mm)	3.86±0.85	$4.00±0.64^{ns}$	$3.52±0.88^{NS}$
LVID;s, (mm)	3.16±0.35	$2.90±0.17^{ns}$	$2.59±0.49^{NS}$
LVAW;d, (mm)	0.77±0.16	$1.01±0.15^{ns}$	$1.10±0.21^{NS}$
LVAW;s, (mm)	1.04±0.17	1.41±0.16**	$1.46±0.22^{NS}$
IVS;d, (mm)	0.93±0.13	$1.12±0.22^{ns}$	$1.13±0.20^{NS}$
IVS;s, (mm)	1.07±0.16	1.45±0.25*	$1.37±0.18^{NS}$

LVID, left ventricular internal diameter; LVAW, left ventricular anterior wall; IVS, interventricular septum; d, diastole; s, systole. ns, represents p>0.05 vs $OTUD5^{fl/y}$ + AAV-NC + Ang II; *, p<0.05 vs $OTUD5^{fl/y}$ + AAV-NC + Ang II; **, p<0.01 vs $OTUD5^{fl/y}$ + AAV-NC + Ang II; NS, represents p>0.05 vs $OTUD5^{fl/y}$ + AAV-NC + Ang II + Stattic.

All data are presented as mean ± SD. For comparisons among more than two groups, Ordinary one-way ANOVA analysis was conducted.

Supplementary Table 10

Echocardiographic parameters of Ang II -challenged and injected with AAV-OTUD5 and STAT3 inhibition mouse experiment at the fourth week

	AAV-NC + Ang II OTUD5CKO n=6 experimental replicates	AAV-NC + Ang II + Stattic OTUD5CKO n=6 experimental replicates	AAV-OTUD5 + Ang II+ Stattic OTUD5CKO n=6 experimental replicates
LVID;d, (mm)	3.82±0.85	$3.97±0.64^{ns}$	$3.72±0.88^{NS}$
LVID;s, (mm)	3.20±0.83	$2.92±0.36^{ns}$	$2.73±0.66^{NS}$
LVAW;d, (mm)	1.07±0.12	$1.04±0.24^{ns}$	$0.92±0.27^{NS}$
LVAW;s, (mm)	1.33±0.15	$1.46±0.28^{ns}$	$1.31±0.29^{NS}$
IVS;d, (mm)	1.08±0.26	$1.12±0.10^{ns}$	$0.98±0.20^{NS}$
IVS;s, (mm)	1.18±0.20	$1.39±0.18^{ns}$	$1.26±0.134^{NS}$

LVID, left ventricular internal diameter; LVAW, left ventricular anterior wall; IVS, interventricular septum; d, diastole; s, systole. ns, represents p>0.05 vs $OTUD5^{fl/y}$ + AAV-NC + Ang II; NS, represents p>0.05 vs $OTUD5^{fl/y}$ + AAV-NC + Ang II + Stattic.

All data are presented as mean ± SD. For comparisons among more than two groups, Ordinary one-way ANOVA analysis was conducted.

<u>Supplementary Table 11</u>

Primary antibodies used in this study

Antibody	Company	Catalog No.	Location
anti-Flag	Proteintech	20543-1-AP	Hubei, China
anti-HA	Proteintech	51064-2-AP	Hubei, China
anti-His	Proteintech	66005-1-IG	Hubei, China
anti-IgG	Proteintech	B900610	Hubei, China
anti-GAPDH	Bioworld	MB001H	Nanjing, China
anti-OTUD5	Proteintech	21002-1-AP	Hubei, China
anti-STAT3	Cell Signaling Technology	12640S	MA, USA
anti-phospho-Tyr705-STAT3	Cell Signaling Technology	9145S	MA, USA
anti-Lamin B	Abcam	ab133741	Cambridge, UK
anti-MyHC	Abcam	ab50967	Cambridge, UK
anti-ANP	Santa Cruz Biotech	SC-515701	Texas, USA
anti-SORBS3	Bai Ao Lai Bo	Z27112	Beijing, China
anti-SPTBN1	Proteintech	19722-1-AP	Hubei, China

Supplementary Fig. 1

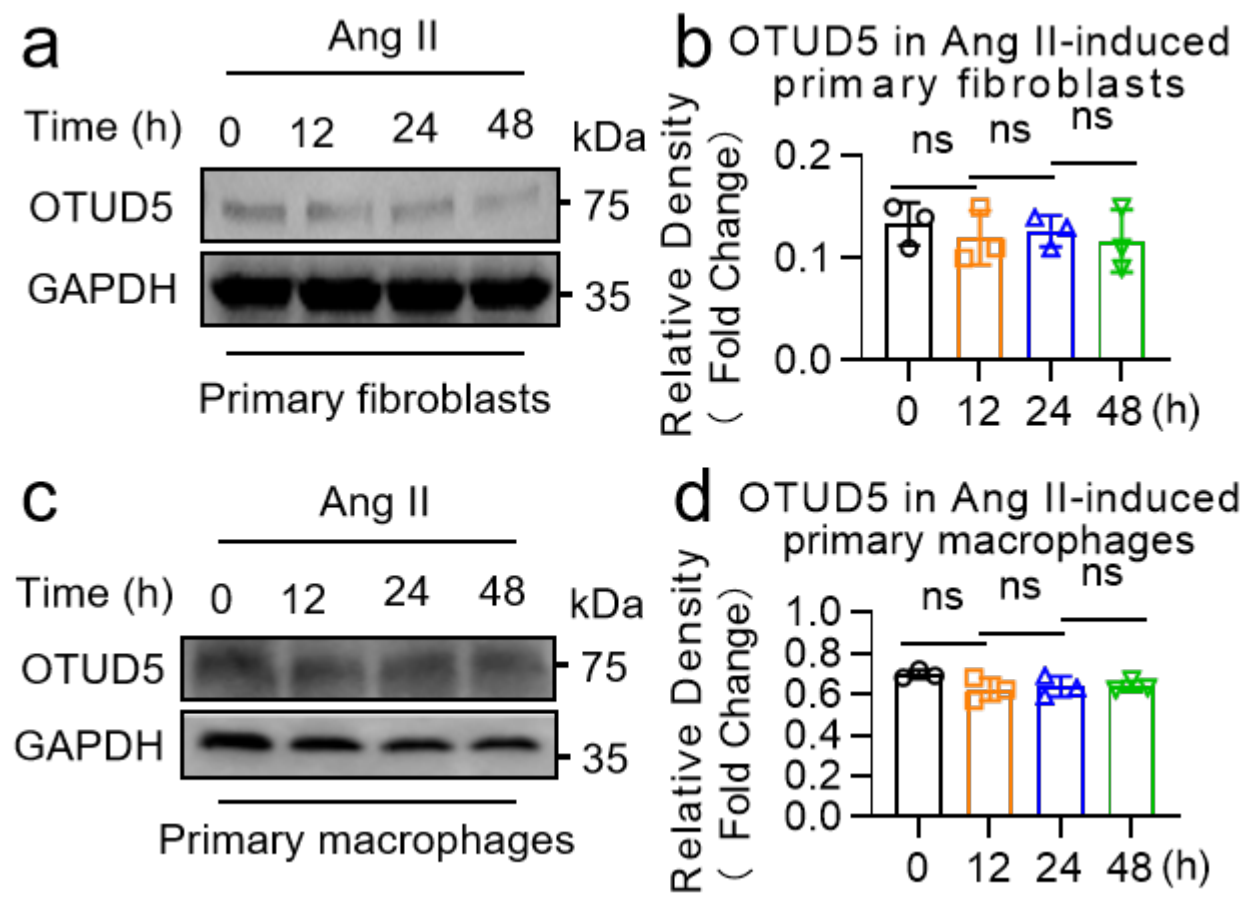

Supplementary Fig. 1. (a–d)OTUD5 expression in primary cardiac fibroblasts (a, b), and primary macrophages (c, d) and the corresponding grayscale intensity analysis. n=3 experimental replicates.

All data are presented as mean ± SD. Between the two groups, t-test was used. For comparisons among more than two groups, Ordinary one-way ANOVA analysis was conducted (b, d). Statistical significance was defined as: **P* < 0.05, ***P* < 0.01, ****P* < 0.001, and *****P* < 0.0001. The abbreviation "ns" indicates no statistical significance (*P* > 0.05).

Supplementary Fig. 2

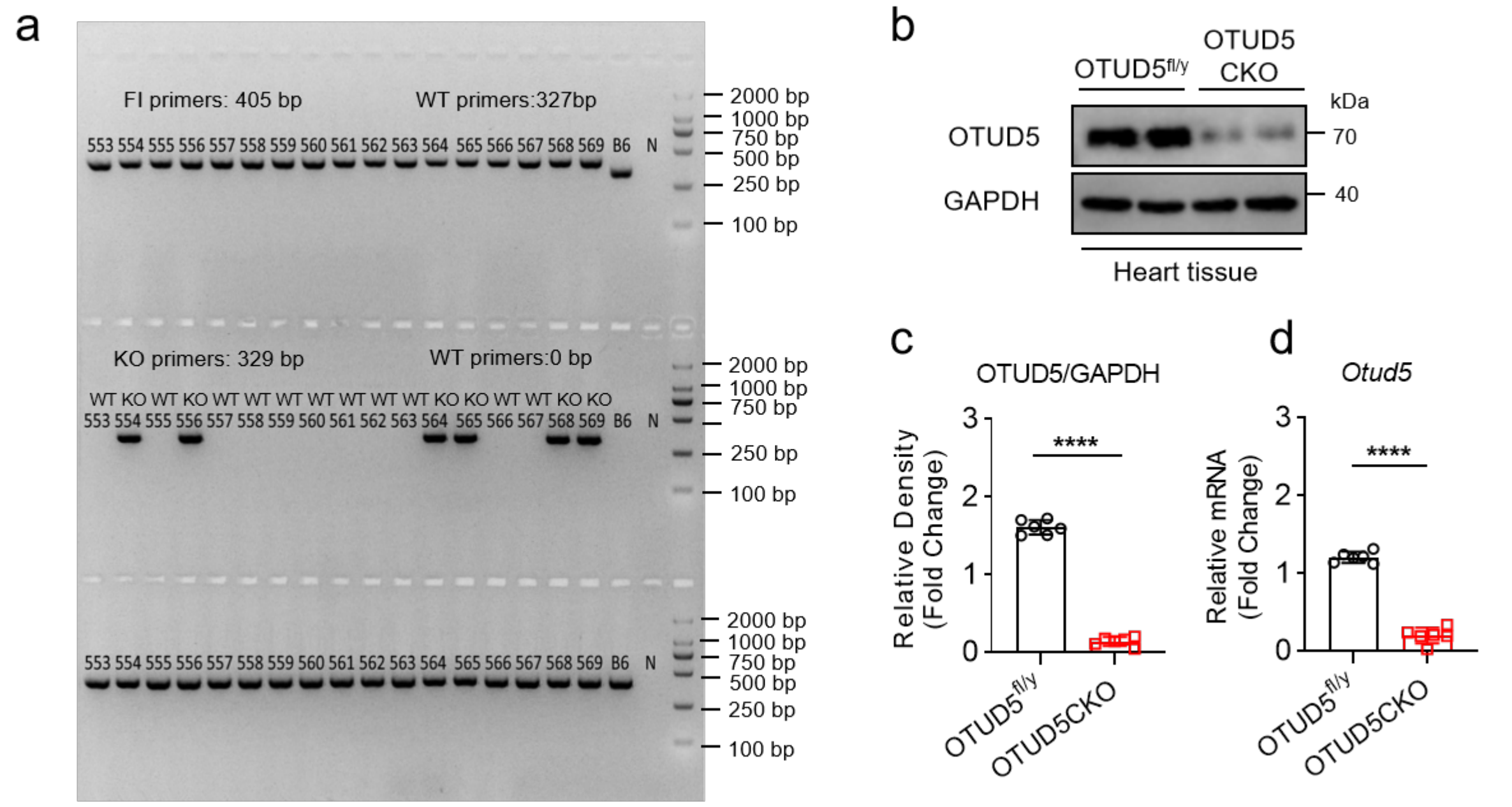

Supplementary Fig. 2. Generation and validation of cardiomyocyte-specific OTUD5 knockout mice.

(a) Genotyping of mouse tail DNA to verify the specificity of OTUD5 knockout using primers 1, 2, and 3. Primer 1 (JS00028-Otud5-5wt-tF1A and JS00028-Otud5-5wt-tR1A) is used to detect the floxed OTUD5 allele (X-linked inheritance), while primers 2 (T004713-F1 and T004713-R1) and 3 (H11-wt-tF1 and H11-wt-tR1) are used to detect the Myh6-Cre transgene. OTUD5$^{fl/y}$ mice exhibit only the bands corresponding to primers 1 (405 bp) and 3 (412 bp). OTUD5CKO mice display bands for primers 1 (405 bp), 2 (329 bp), and 3 (412 bp).

(b–c) Western blot analysis of OTUD5 protein expression in myocardial tissues of OTUD5$^{fl/y}$ and OTUD5CKO mice, along with a corresponding bar graph showing quantitative results. n = 6 experimental replicates.

(d) Real-time quantitative PCR (qPCR) analysis of OTUD5 mRNA levels in myocardial tissues of OTUD5$^{fl/y}$ and OTUD5CKO mice. n = 6 experimental replicates.

All data are presented as mean ± SD. Between the two groups, t-test was used. Statistical significance was defined as: *P < 0.05, **P < 0.01, ***P < 0.001, and ****P < 0.0001. The abbreviation "ns" indicates no statistical significance (P > 0.05). n=6 experimental replicates.

Supplementary Fig. 3

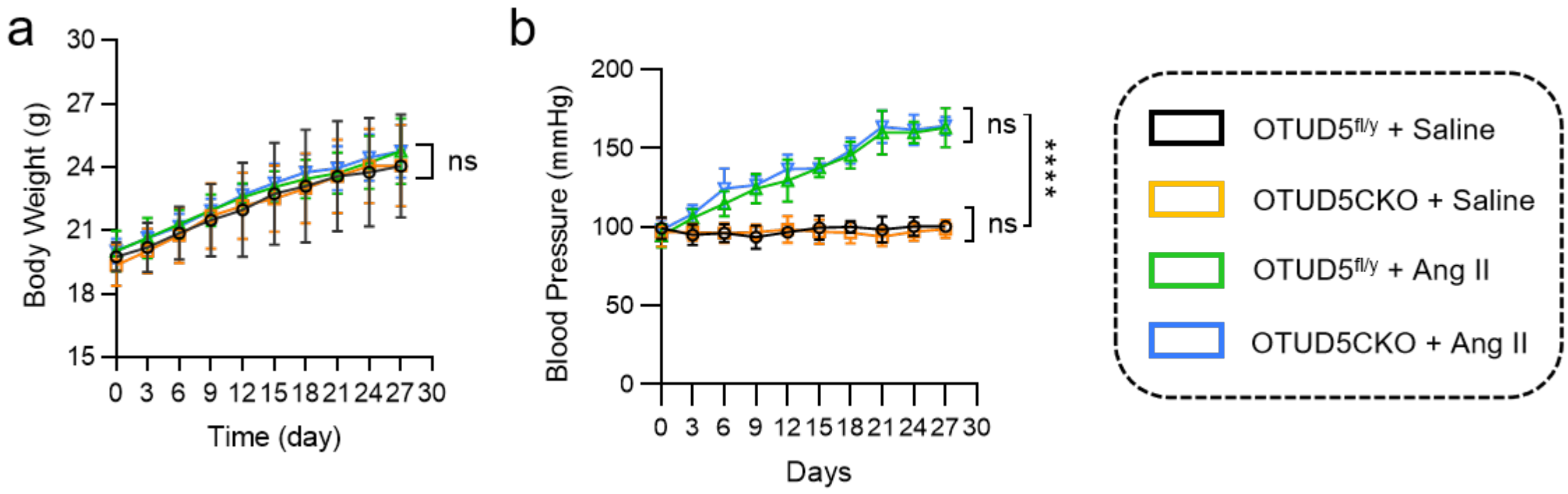

Supplementary Fig. 3. OTUD5CKO and OTUD5$^{fl/y}$ mice were subcutaneously implanted with osmotic mini-pumps delivering either saline or Ang II (1000 ng/kg/min) for 4 weeks.

(a) The weight of mice in each group. n=6 experimental replicates.

(b) The blood pressure of the tail artery in each group of mice. n=6.

All data are presented as mean ± SD. For comparisons among more than two groups, Ordinary one-way ANOVA analysis was conducted. Statistical significance was defined as: ****P< 0.0001, OTUD5$^{fl/y}$ + Saline v.s. OTUD5$^{fl/y}$ + Ang II; The abbreviation "ns" indicates no statistical significance (P > 0.05).n=6 experimental replicates.

Supplementary Fig. 4

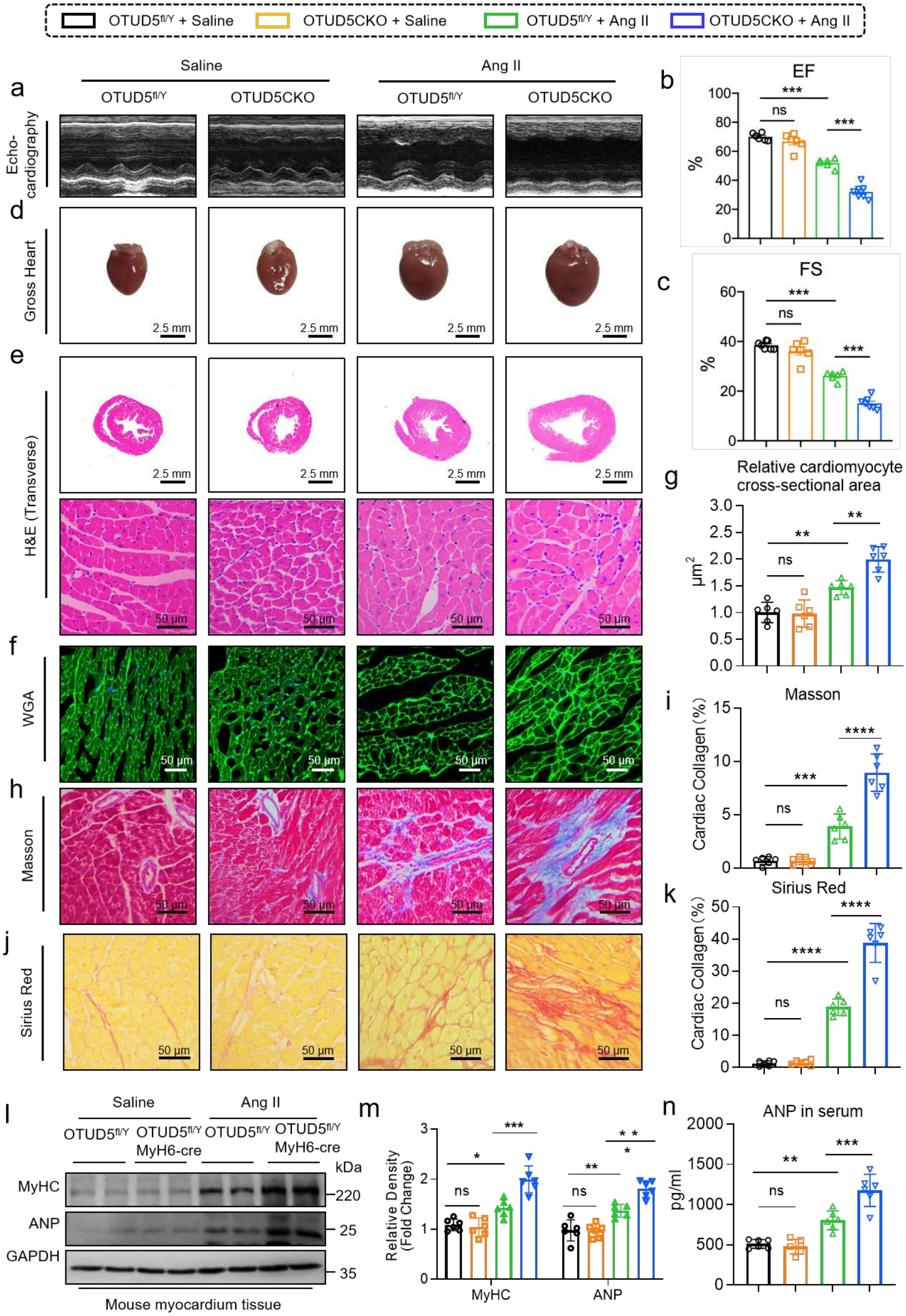

Supplementary Fig. 4. Cardiomyocyte-specific deficiency of OTUD5 exacerbates Ang II-induced cardiac dysfunction and myocardial hypertrophy. OTUD5CKO and OTUD5$^{fl/y}$ mice were subcutaneously implanted with osmotic mini-pumps delivering either saline or Ang II (1000 ng/kg/min) for 4 weeks.

(a–c) Representative M-mode echocardiography of mice (a), ejection fraction (EF, b) and fractional shortening (FS, c) were evaluated in mice through echocardiography.

(d) Representative gross heart images. Scale bar = 2.5 mm.

(e) Representative H&E-stained heart sections. Scale bar = 2.5 mm.

(f-g) Representative WGA-stained sections (f) and quantitative analysis of cardiomyocyte cross-sectional area (g). Scale bar = 50 μm.

(h-i) Representative Masson's trichrome-stained sections (h) and quantitative analysis of fibrotic area (i). Scale bar = 50 μm.

(j-k) Representative Sirius Red -stained sections (j) and quantitative analysis of fibrotic area (k). Scale bar = 50 μm.

(l-m) Protein levels of MyHCand ANP (l) in mouse heart tissues and quantitative analysis (m).

(n) Serum ANP levels in mice.

All data are presented as mean ± SD. For comparisons among more than two groups, Ordinary one-way ANOVA analysis was conducted. Statistical significance was defined as: *P < 0.05, **P < 0.01, ***P < 0.001, and ****P < 0.0001. The abbreviation "ns" indicates no statistical significance (P > 0.05). n=6 experimental replicates.

Supplementary Fig. 5

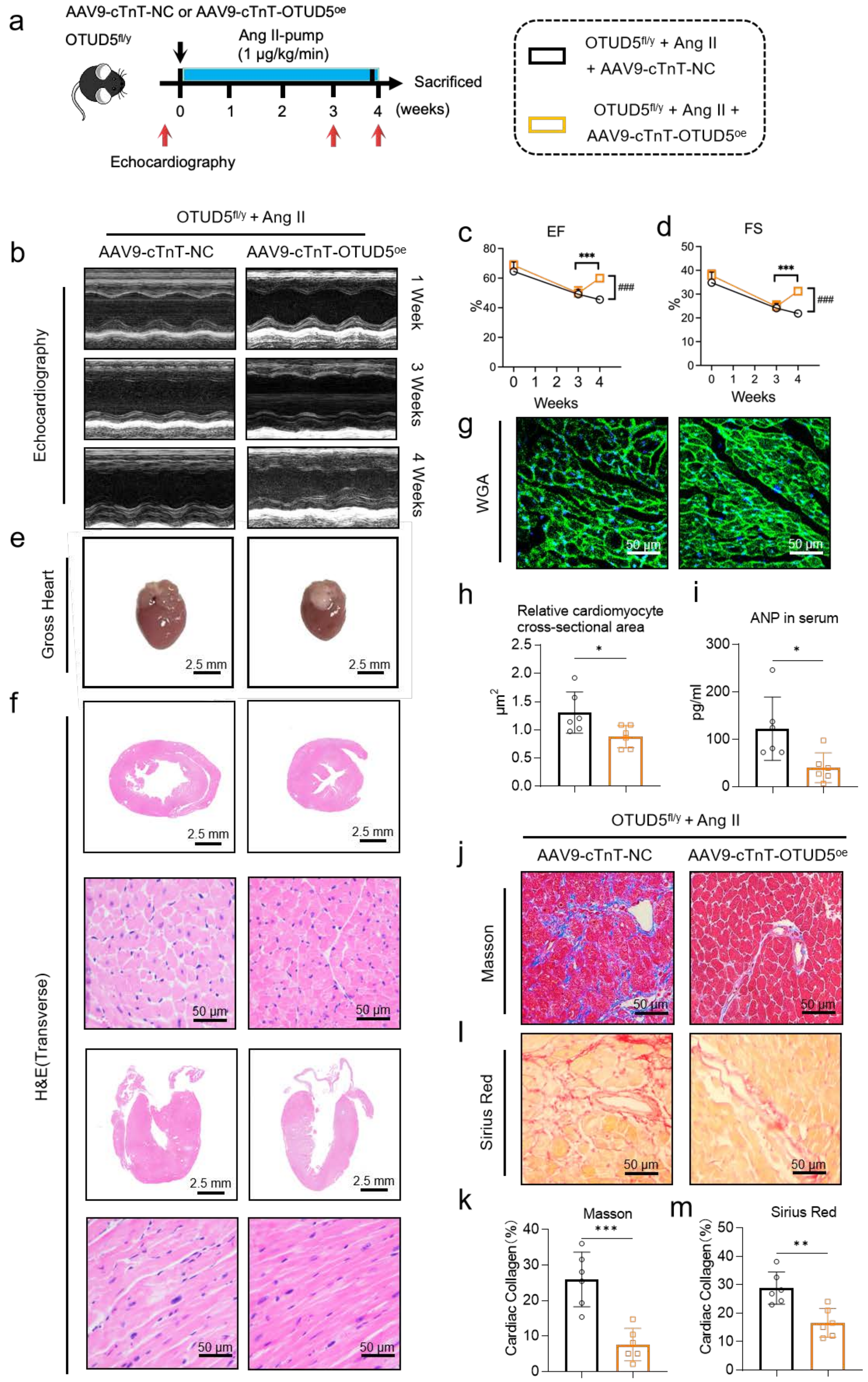

Supplementary Fig. 5. Cardiomyocyte-specific overexpression of OTUD5 attenuates Ang II-induced cardiac hypertrophy and dysfunction. OTUD5CKO mice received tail vein injections of either AAV9-cTnT-NC or AAV9-cTnT-OTUD5, followed by subcutaneous implantation of

osmotic minipumps delivering either saline or angiotensin II (Ang II, 1000 ng/kg/min) for 4 weeks. Cardiac function assessments were performed at 1-, 3- and 4-weeks post-treatment initiation.

(a) Schematic illustration of the experimental model establishment.

(b–d) Representative M-mode echocardiography of mice (b), ejection fraction (EF, c) and fractional shortening (FS, d) were evaluated in mice through echocardiography.

(e) Representative gross heart images. Scale bar = 2.5 mm.

(f) Representative H&E-stained heart sections. A gross transverse or longitudinal incision of the heart, Scale bar = 2.5 mm. Enlarged sections of the heart, Scale bar = 50 μm.

(g-h) Representative WGA-stained sections (g) and quantitative analysis of cardiomyocyte cross-sectional area (h). Scale bar = 50 μm.

(i) Serum ANP levels in mice.

(j-k) Representative Masson's trichrome-stained sections (j) and quantitative analysis of fibrotic area (k). Scale bar = 50 μm.

(l-m) Representative Sirius Red -stained sections (l) and quantitative analysis of fibrotic area (m). Scale bar = 50 μm.

All data are presented as mean ± SD. Between the two groups, t-test was used. Statistical significance was defined as: *P < 0.05, **P < 0.01, ***P < 0.001, and ****P < 0.0001. The abbreviation "ns" indicates no statistical significance (P > 0.05). n=6 experimental replicates.

Supplementary Fig. 6

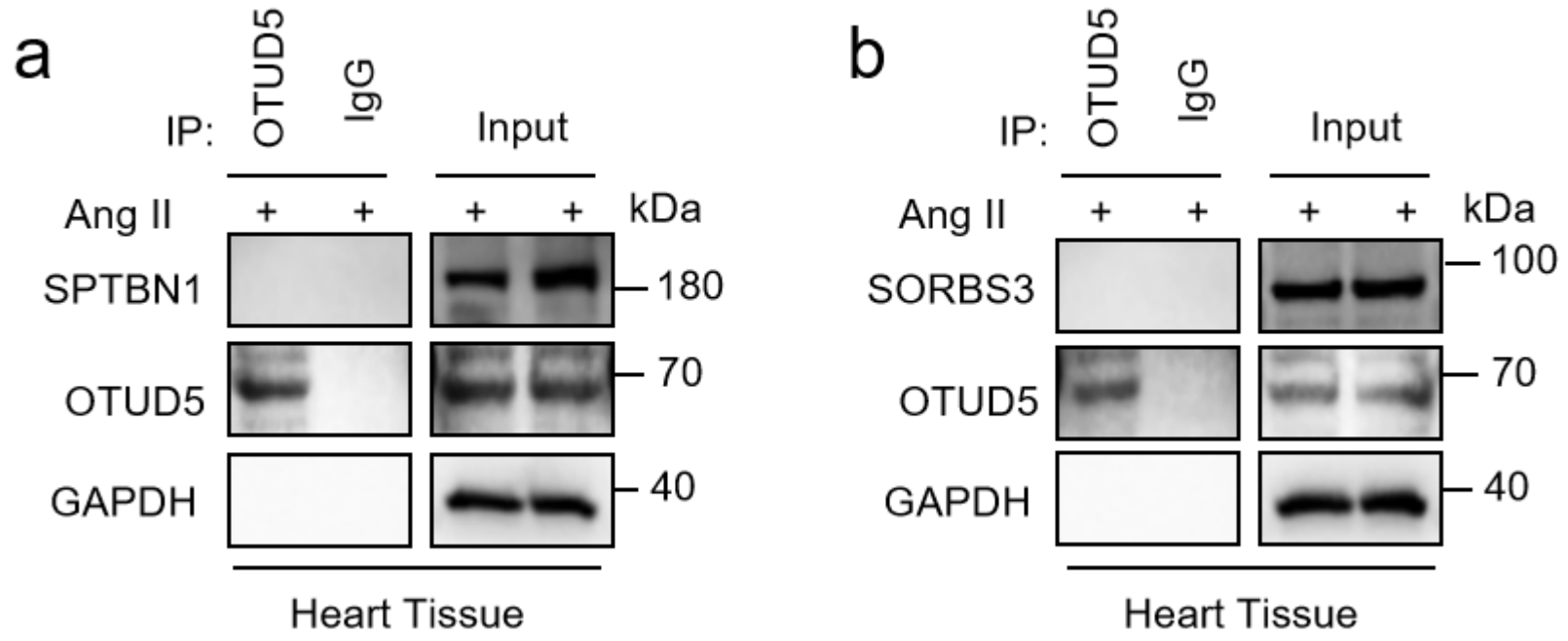

Supplementary Fig. 6.
(a) Co-IP of OTUD5 and SPTBN1 in heart tissue lysates
(b) Co-IP of OTUD5 and SORBS3 in heart tissue lysates

Supplementary Fig. 7

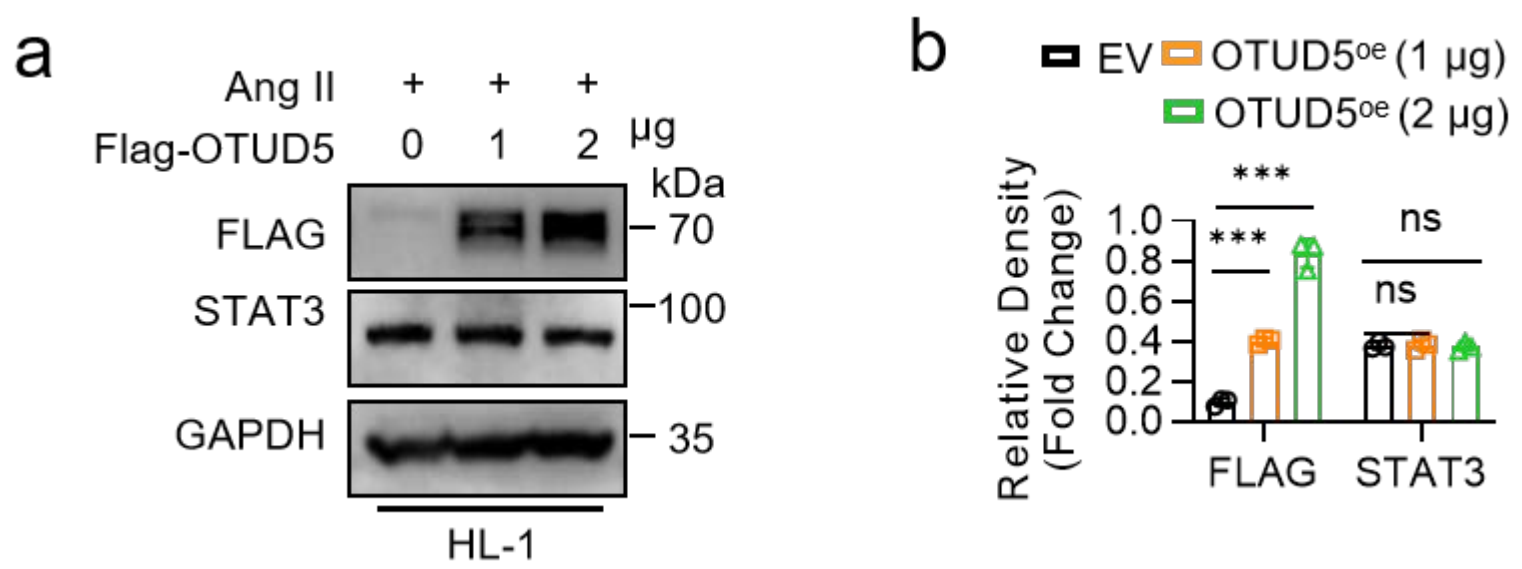

Supplementary Fig. 7. NIH/3T3 cells were overexpressed with 0, 1, and 2 μg Flag-OTUD5 plasmid and stimulated for 24 hours with Ang II (10 μM).
(a-b) Western blotting analysis of Flag-OTUD5 and STAT3 protein levels and corresponding gray-scale value statistical charts. All data are presented as mean ± SD. For comparisons among more than two groups, Ordinary one-way ANOVA analysis was conducted. Statistical significance was defined as: *P < 0.05, **P < 0.01, ***P < 0.001, and ****P < 0.0001. The abbreviation "ns" indicates no statistical significance (P > 0.05). n=3 experimental replicates.

<u>Supplementary Fig. 8</u>

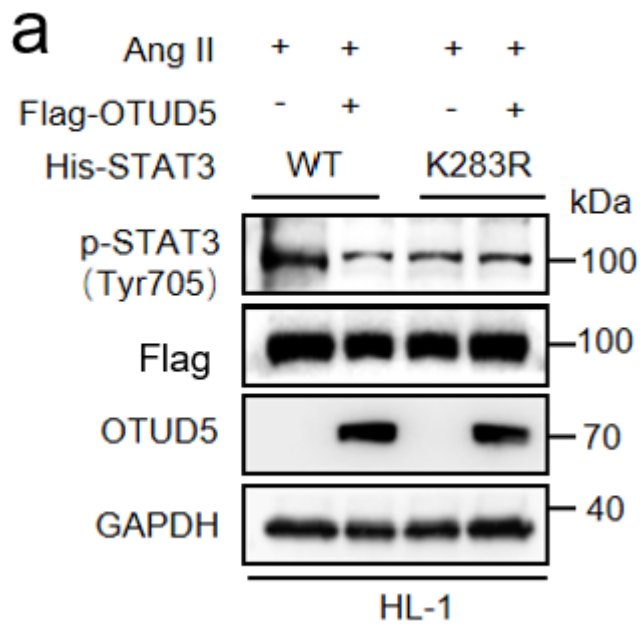

Supplementary Fig. 8. HL-1 cells were overexpressed with Flag-OTUD5 plasmid and His-STAT3 (WT or K283R) plasmid stimulated for 24 hours with Ang II (10 μM).

(a) Western blotting analysis of Flag-OTUD5 , STAT3 protein levels and p-STAT3(Tyr705) protein levels.

Supplementary Fig. 9

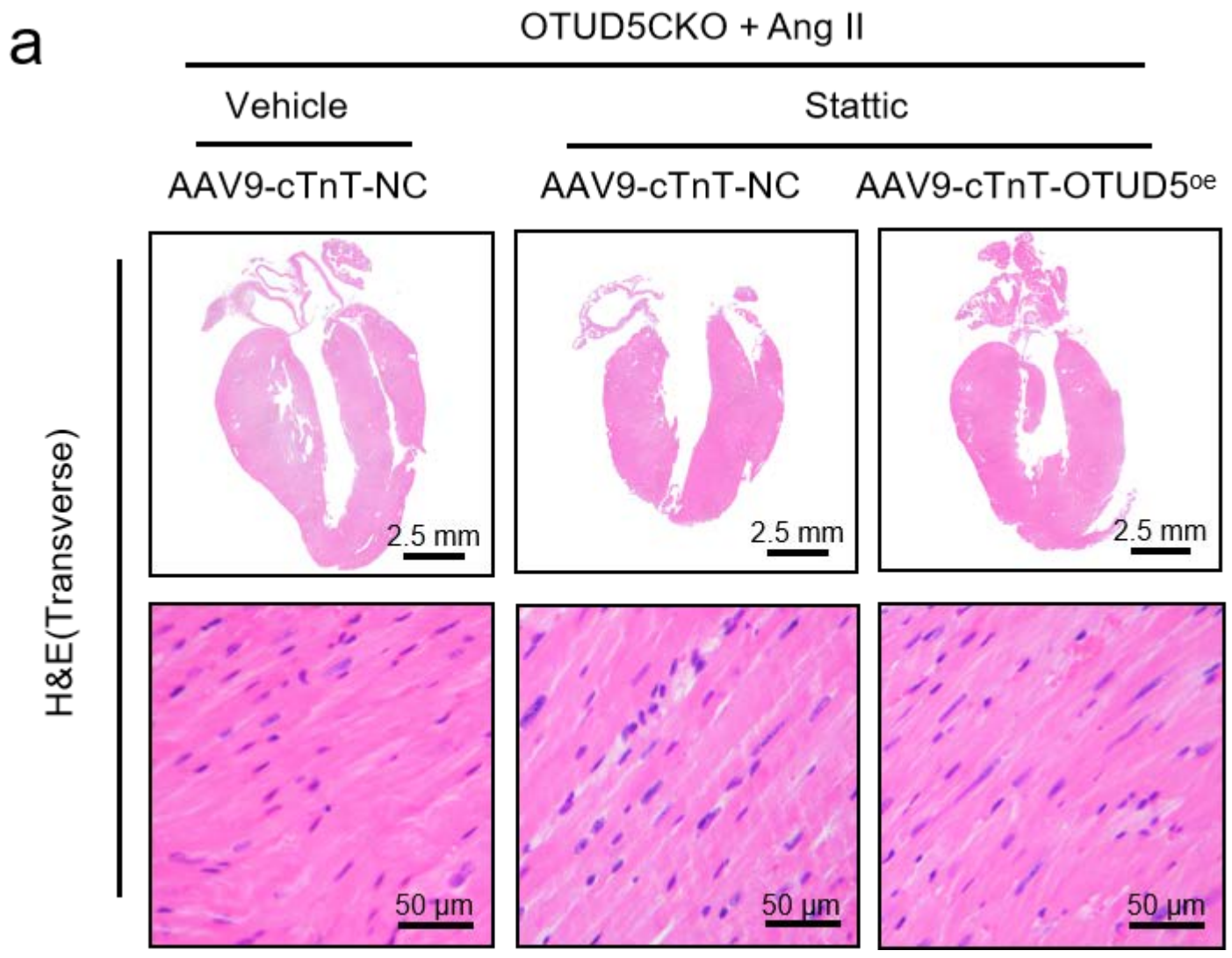

Supplementary Fig. 9. OTUD5CKO mice received tail vein injections of either AAV9-cTnT-NC or AAV9-cTnT-OTUD5, followed by subcutaneous implantation of osmotic mini-pumps delivering either saline or Ang II (1000 ng/kg/min) for 4 weeks. During the 4-week treatment period, mice were orally administered either the STAT3-specific inhibitor Stattic (10 mg/kg) or vehicle control via gavage. Cardiac function assessments were performed at weeks 1, 3, and 4.

(a) Representative H&E-stained heart sections. The gross heart, Scale bar = 2.5 mm. Enlarged sections of the heart, Scale bar = 50 μm.
